# The role of microRNAs in executive functions: a comprehensive review and bioinformatics analysis of human and animal studies

**DOI:** 10.1038/s41380-026-03623-2

**Published:** 2026-05-01

**Authors:** Alba Navarro-Flores, Dennis M. Krüger, Lalit Kaurani, Andre Fischer, Thomas G. Schulze, Urs Heilbronner

**Affiliations:** 1 Institute of Psychiatric Phenomics and Genomics (IPPG), LMU University Hospital, LMU Munich, Munich, Germany; 2 International Max Planck Research School for Translational Psychiatry (IMPRS-TP), Munich, Germany; 3 Max-Planck-Institute of Psychiatry, Munich, 80804 Germany; 4 Department for Epigenetics and Systems Medicine in Neurodegenerative Diseases, German Center for Neurodegenerative Diseases, Göttingen, Germany; 5 Laboratory of Neuroepigenetics, Institute of Physiology of the Czech Academy of Sciences, Videnska 1083, 14200 Prague, Czech Republic; 6 Cluster of Excellence “Multiscale Bioimaging: from Molecular Machines to Networks of Excitable Cells” (MBExC), University of Göttingen, Göttingen, Germany; 7 Department of Psychiatry and Psychotherapy, University Medical Center Göttingen, Göttingen, Germany; 8 Department of Psychiatry and Behavioral Sciences, SUNY Upstate Medical University, Syracuse, NY USA; 9 Department of Psychiatry and Behavioral Sciences, Johns Hopkins University School of Medicine, Baltimore, MD USA; 10 German Center for Mental Health (DZPG), partner site Munich-Augsburg, Munich, Germany

**Keywords:** Genetics, Diagnostic markers

## Abstract

Executive functions (EFs) are meta-cognitive abilities that orchestrate goal-directed behavior (i.e., set-shifting, working memory, and inhibitory control). Despite their strong genetic composition, the development of EFs is shaped by environmental exposures – as maternal distress, perinatal hypoxia, household dysfunction, neglection - which have varying degrees of impact depending on the duration or severity of the exposure, sensitive neurodevelopmental periods, and individual resiliency. Furthermore, they are negatively affected by aging and psychiatric, neurologic, and inflammatory diseases. MicroRNA dysregulation interferes with normal brain development and function and has been associated with various neuropsychiatric disorders; however, its effects on EFs remain unclear. Therefore, in this review we focus on the evidence regarding microRNA changes and their effects on EFs. We performed a systematic search from inception until October 2023 of four databases of human and animal studies. The results are presented narratively. Moreover, we conducted a bioinformatics analysis using experimental mRNA targets of the candidate microRNAs, as well as assessed the risk of bias of the included studies. We found 46 studies (23 in humans, 22 in animals, and one in both). The studies evaluated mild cognitive impairment, psychiatric disorders, and healthy aging. Gene mutations in MIR137 were associated with decreased EF performance, whereas MIR885 gene methylation was associated with increased executive functioning. Mutations in the genes of enzymes relevant for microRNA biosynthesis also impacted EFs. In the revised literature, the microRNAs that were consistently reported as dysregulated in two or more samples in relation to variations in EFs were miR-148a-3p for humans; miR-155, miR-30e, and miR-384-5p for rodents; and miR-132, miR-146a-5p, miR-148-3p, miR-181a-5p, miR-190b, miR-31, miR-501-3p, and miR-9-5p for both humans and rodents. The suggested regulatory pathways behind EFs included changes in neurogenesis, neurodevelopment, and synaptic plasticity/signaling. Changes in the various steps of microRNA biogenesis - such as mutations in the genes coding for microRNAs, reduced availability of relevant processing enzymes, or dysregulation of microRNA expression - are potentially associated with changes in EFs. Future research is required to better understand these associations in relation to developmental stage, diagnosis, disease severity, and the degree of the microRNA dysregulation.

## Introduction

Executive functions (EFs) are high-level cognitive processing skills that are relevant for self-regulation and goal-directed behaviors. [1] They support task initiation, performance, persistence, and completion and are therefore indispensable for occupational activities. [2] Some core abilities comprised under the umbrella term EFs are set-shifting (i.e., alternating between tasks), working memory (i.e., holding on to relevant information for relatively short periods of time), and inhibitory control (i.e., preventing the manifestation of an inappropriate cognitive process or behavior). [3] Although these abilities are separately identifiable, they share a top-level latent component named the common EF factor that explains most of the variability across them. [1]

EFs are presumed to be the result of the dynamic interplay between inherited factors and environmental influences, although they are also subject to independent influences of genes and environment separately. [4] The genetic architecture behind EFs is responsible for their high heritability and has been extensively studied. For example, twin studies estimated the heritability in children and adolescents to be as high as 0.77–1.00, whereby the latter value was obtained when researchers used the common EF factor. [5] Executive functioning involves activation of different neurotransmitters and circuits, including the serotoninergic, dopaminergic, and cholinergic systems, and changes in the genes related to the biosynthesis or transportation of these molecules are related to impairment of EFs. [6, 7] Recently, Hatoum et al. conducted the largest genome-wide association study (GWAS) on EFs to date. The study, which included more than 400 000 individuals in the UK biobank, found that genes related to inhibition (i.e., GABAergic processes) mediated EFs and also identified opposing genetic correlations between EFs and psychiatric disorders. [8]

Environmental adversities throughout the neurodevelopmental landscape contribute further to the shaping of EF. [9] The consequences of these exposures depend on various factors as their duration or severity, sensitive neurodevelopmental periods, and individual resiliency. Some examples include prenatal exposure to maternal distress, substance use or psychiatric disorders, perinatal hypoxia-ischemia, and adverse childhood experiences (ACEs). It is known that late childhood and adolescence are critical periods for the development of EFs, [10] therefore ACEs as physical abuse, sexual abuse, emotional or physical neglect during these periods of life can negatively impact EFs (mostly overall measures or working memory) into early and middle adulthood, and in the absence or presence of additional psychopathology (i.e., patients with bipolar disorder or schizophrenia). [9, 11] In adulthood, aging has mostly negative effects on EFs, although inhibitory control may increase with age. [12] EFs are also affected by chronic diseases, including cardiovascular and metabolic conditions. [13] Importantly, lower EF scores are found in most psychiatric diseases, including major depressive disorder (MDD), [14] bipolar disorder, [15] schizophrenia, [16] and post-traumatic stress disorder. [17] EFs are moldable skills and susceptible to improvement by cognitive remediation interventions, which are additionally effective for negative symptoms and overall functioning in patients with schizophrenia, in whom greater symptom severity and later onset-age do not prevent gains. [18, 19] Furthermore, meta-analyses showed that cognitive remediation is effective for improving EFs in adults with MDD, [20] and reducing symptoms severity and enhancing working memory in patients with anxiety disorders. [21] In summary, EFs are affected by genetic and environmental factors and are highly plastic and sensitive to rehabilitation.

**Fig. 1:**
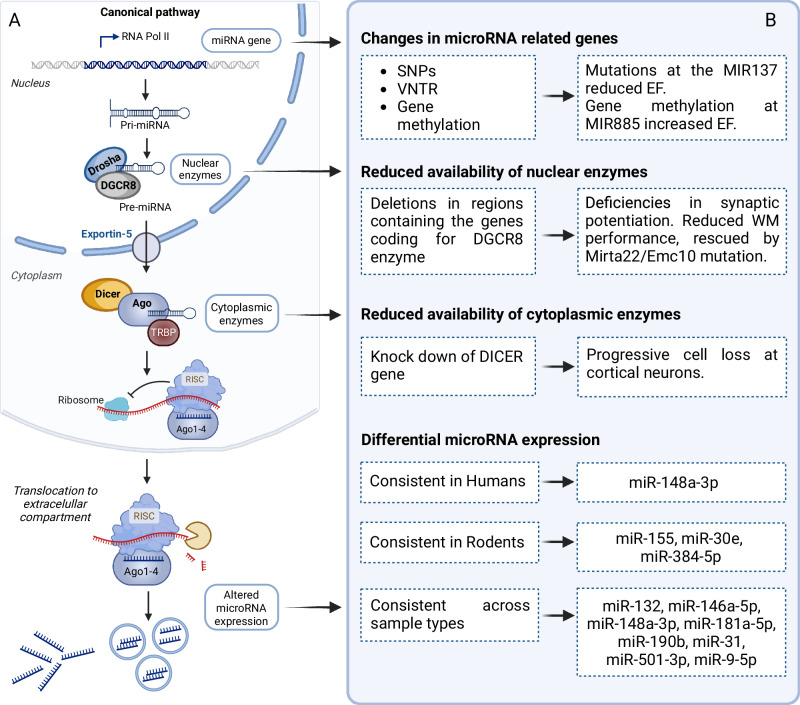
Stages of microRNA biogenesis and their disruption in relation to executive functions. **A** Canonical pathway of microRNA biosynthesis. **B** Summary of the findings according to each step of the synthesis process. SNP, single nucleotide polymorphism; VNTR, variable number tandem repeat. Created in BioRender. Navarro Flores, A. (2026) https://BioRender.com/ztbg5cd. Partially adapted from Huang, E. [29], O’Carroll et al. [28], and Maffioletti et al. [27–29].

Epigenetic modifications have a profound impact on the genome and increase or decrease the expression of genes. [22] Examples of such modifications include DNA methylation, histone modification, and regulation by non-coding RNAs, which include long non-coding RNAs and microRNAs. MicroRNAs are short non-coding sequences of around 22 nucleotides that were originally discovered in the genome of C. elegans three decades ago [23]; they participate in the silencing of mRNAs [24] and are assumed to be able to directly or indirectly modulate the expression of around a third of protein-coding genes. [25] The biosynthesis of microRNAs starts with the transcription of the corresponding gene. This primary microRNA is then processed by the nuclear enzymes DGCR8 and Drosha (which together form the microprocessor complex) to produce a precursor microRNA. The resulting sequence is translocated to the cytoplasm by exportin 5 and then further processed by the cytoplasmic enzyme DICER and by AGO proteins. The mature microRNA can exert effects in the cell or surrounding cells and can also reach distant tissues via the peripheral circulation, and the main overall effect of microRNAs is the regulation of gene transcription (Fig. 1A) [26–29]. Alterations in any of the relevant steps of microRNA biosynthesis can alter microRNA expression. In addition, microRNAs can target hundreds to thousands of mRNAs, being capable of regulating multiple genes and different individual biological mechanisms simultaneously leading to high functional pleiotropy of their effects, especially in complex systems as the nervous system. [30]

MicroRNAs are enriched in the brain in a tissue-specific manner. [31] They are expressed in various areas relevant for executive functioning, such as the frontal cortex and hippocampus, and are involved in crucial processes, such as neural genesis and differentiation, and synapse formation [32]. Therefore, dysregulation of microRNA expression due to changes in their biosynthesis or a mutation in the responsible gene can potentially induce various brain-related disorders [33]. For example, deletion of the region 22q11.2, which is critical for expression of the DiGeorge complex protein (a protein responsible for maturation of the microRNA), produces DiGeorge Syndrome; individuals with this deletion present with neurodevelopmental difficulties (learning disabilities) and a high prevalence of schizophrenia (25%) and other psychiatric disorders [34]. This example shows the importance of proper microRNAome expression for mental health. Moreover, many studies have reported dysregulated expression of microRNAs as potential biomarkers for certain psychiatric disorders [35–37], neurodegenerative disorders [33, 38], and chronic inflammatory conditions [39]. Establishing microRNAs as biomarkers for EFs would also be advantageous because microRNAs found peripherally (i.e., in blood samples) are informative of brain processes, highly stable in cell-free environments, and resistant to thaw-freeze cycles and could represent targets for future RNA-based therapies [40–42].

EFs are fundamental for regulating behavior, cognition, and mental health [43]. They decline not only in patients with psychiatric and neurologic disorders, but also in patients with chronic inflammatory diseases and during normal aging [44, 45]; while disturbances of EFs produce significant burden and impairment in activities of daily living and quality of life [46]. Understanding the role of microRNA expression represents an opportunity to better comprehend the epigenetic mechanisms involved. Furthermore, it may indicate targets that—with further research—could generate novel noninvasive biomarkers and potential therapeutic options. Here, we synthesize the evidence from published studies on the association between microRNA dysregulation and EFs in both human cohorts and animal models.

## Methods

### Literature search

This comprehensive literature review was performed according to the PRISMA guidelines [47]; a checklist of the items used is presented in the Supplement **(**eTable 1**)**.

We searched the databases PubMed, WoS, and Scopus from inception until October 2023 by using the key terms “executive functions,” “working memory,” “Trail Making Test,” “microRNA,” and “miRNA” with Boolean connectors. The full search terms are presented in the Supplement **(**eTable 2**)**. The inclusion criteria were as follows: 1) original article on observational (e.g., cross-sectional, case control, or cohort) or experimental study in humans or animals; 2) assessed EFs as a group of abilities or as individual skills such as working memory, set-shifting, and inhibitory control; and 3) assessed microRNA-related biology (e.g., microRNA expression, enzymes involved in microRNA synthesis, genes that regulate microRNA expression) and calculated correlations with executive functioning (i.e., explicitly stated that EFs were studied in general or named any individual EFs, including but not limited to working memory, set shifting, and inhibitory control). We excluded conference abstracts, studies on humans with cancer or animal models of cancer, studies in which the outcome of the microRNA analysis was not related to EFs (e.g., microRNA dysregulation in depression), and studies that analyzed only cognitive measures that were not EFs. No restrictions were placed on language, participant age, or publication time. One researcher (AN) screened the title and abstracts of the articles; in articles that appeared to be eligible for inclusion, this author read the full text, revised the inclusion criteria, and determined the final decision of inclusion. A second reviewer (UH) intervened in case of queries.

### Quality assessment

The quality of the included human and animal studies was evaluated separately. It was assessed by one author (AN), and discrepancies were solved by a second author (UH). For the human studies, we used the New Castle Ottawa tool [48] to evaluate case-control and cohort studies and adapted the tool for use with cross-sectional studies. For randomized controlled trials, we used the revised Cochrane risk-of-bias tool (RoB 2.0) [49]. The animal studies were assessed with the SYRCLE RoB tool [50], and the criteria were adapted to our research question in a similar way as used previously [51].The criteria are described in detail in the Supplement **(**eTables 3–6**)**.

### Synthesis of the evidence

The results are summarized as a narrative review, and the information on the human and animal studies is presented separately, both in the text and in the tables. Figures were created using Biorender [52].

### Bioinformatics analysis

MicroRNAs were included if they were reported as being significantly dysregulated, the dysregulation correlated with impairment of EFs, and if they were found in experimental studies. We analyzed the human- and animal-derived candidates separately. Bioinformatics included all miRNAs independent of their brain expression status, [53] as peripheral miRNAs can influence brain function via direct crossing of the blood-brain-barrier or transport in extracellular vesicles. [40, 54]

The following four groups were created by using the direction of the microRNA regulation (i.e., up or down) and the associated effect on EFs (i.e., increased or decreased): Group 1, microRNA upregulated and EFs increased; Group 2, microRNA upregulated and EFs decreased; Group 3, microRNA downregulated and EFs increased; and Group 4, microRNA downregulated and EFs decreased.

### Inclusion criteria

#### Context and tissue specificity

When analyzed in similar tissues and stages of development, the expression and biological effects of many microRNAs are conserved across mammals [55]; however, although rare, major differences can occur in expression and directionality of effect between species. [56] These differences could reflect species-specific microRNA profiles, especially in brain tissue and in certain environmental situations or disease models, and consequently warrant further investigation. [56, 57] Therefore, we analyzed human and animal studies separately and tolerated contradictory results between these two sample types.

#### Functional consistency

Functional consistency was the inclusion criterion for the studies that reported microRNA results within the same sample type (i.e., human or rodents). MicroRNA functional consistency is present when similar regulatory effects (in this case, increased or decreased EFs) are associated with regulation in the same direction (i.e., up or down). Consistency was also accepted when a correlation was reported in both logical directions, e.g., when upregulation of one microRNA was associated with higher EF performance and downregulation of the same microRNA was associated with lower EF performance. [58, 59] Moreover, if a correlation was found in one direction but not in the other (e.g., upregulation was associated with higher EF performance, but downregulation was not associated with changes in EFs) it was not assumed to inherently reflect inconsistency because unidirectional effects can be observed when functional threshold effects, [60, 61] molecular titration, [62] or redundant or compensatory roles of other regulatory molecules come into play. [63]

MicroRNAs that did not fulfill the above criteria within a sample type were considered inconsistent and excluded from the bioinformatics analysis. MicroRNAs that were reported as not having any human homology or were not annotated in the tool, as well as those from studies with models of alcohol abuse or negative behavioral results were also not included.

### Network and gene ontology analysis

The targets of these microRNAs (coding genes) were identified using an in-house tool (created by DK & AF), as previously published [64]. The tool integrates exclusively experimentally validated targets collected from six different databases (NPInter [65], TarBase [66], TransmiR [67], RegNetwork [68], Rise [69], and STRING [70]). From those we selected the mRNA targets that were enriched in brain tissue (expression two-fold higher in brain) using the RNA Atlas [71], and gene ontology analyses were performed acknowledging the probabilistic nature of predictions. We included the biological processes section of the enrichGO function, by using a gene set enrichment analysis with false discovery rate correction (clusterProfiler package [72], R v.4.4.1 [73]).

A network analysis of these targets was performed with Cytoscape V.3.7.2. [74] We ranked nodes by degree centrality and, reported the top 10 hubs per group as an a priori decision to provide a concise yet representative subset of the most connected genes as an overview for biological interpretation.

## Results

### Overview of the literature search results

A total of 590 records were retrieved, 102 were further full-text assessed, and finally 46 studies were included. From them, 23 were studies only in humans, [75–97] 22 were studies only in animals, [98–119] and one was a study in both humans and animals [40]. Only animal studies in rodents were found, therefore the terms will be used interchangeably. A detailed description of the included human and animal studies is presented in Table 1 and Table 2, respectively. The selection process is summarized in eFigure 1, and a list of the excluded studies, together with the reasons for exclusion, is presented in eTable 7.

**Table 1:** Characteristics of the human studies.

Study	Country	Diagnosis/Condition	Sample size	Mean age^┼^	Education years (mean)	Design	EF tests	Sample source	MiRNA Detection Method	Analysis of microRNA expression
Anderson-Hanley [97]	USA	MCI (Diagnosed or at high risk)	14 (after dropouts, 111 enrollees)	78.1	16.2	RCT	Stroop test, Color trails, and Digit span	Exosomes from saliva	miRNA-193 and miRNA-9, detected using real-time quantitative PCR (qRT-PCR), normalized to U6.	Partial correlations controlling for previously related variables like age and sex.
Andrews [96]	Australia	Aging	1570	62.5	14	Prospective cohort	Digit Span Backward from the Wechsler Memory Scale (WMS)	NR	Genotyping of SNPs reported in previous GWAS (MIR2113-rs10457441) using TaqMan OpenArray Assays.	Adjusted (gender and APOE genotype) linear mixed effects models were estimated, including longitudinal cognitive changes, using the R package ‘lme4’.
Aparicio [95]	Canada	HIV	33	49.1	13.9	Cross-sectional	TMT-4, and Wisconsin Card Sorting Test (WCST)	Plasma, circulating microRNAs	A broad screening of a total of 3391 different miRNAs, using qRT-PCR and miR-16.5p for normalization.	A random forest (RF) machine learning model was fitted using the z-scores of the cognitive tests to predict important miRNAs (controlling for sex, years of education, CD4 nadir, and viral load).
Bijkerk [94]	Netherlands	End-stage renal disease	129	75.3*	Higher education in 27.1% (n = 35)	Cross-sectional analysis of prospective cohort	Trail Making Test A and B (TMT-A and -B), Stroop Test	Plasma	Specific miRNAs levels detected with reverse transcription qPCR, normalized to stable miR-16 (miRCURY set).	Associations were assessed using linear regression analyses, adjusted for age and gender.
Cosgrove [93]	Ireland	Schizophrenia	1000 (808 cases, 192 HC)	41.4	NR	Case-controls	Spatial Working Memory subtest from the CANTAB, and Letter-Number Sequencing from the WMS	Whole blood or saliva	Full genome-wide SNP genotypification. Then, genes downstream related to microRNA-137 expression were analyzed.	Polygene scores were calculated using miR-137-related downstream genes. The two samples were analyzed separately using linear regression and then meta-analyzed. Effect sizes were reported with r^2^ and standardized beta values.
Dypås [92]	UK, France, Spain, Lithuania, Norway, and Greece	ADHD	1126	8.2*	Maternal education: low 15.5%, medium 32.4%, high 46.7%, missing data 5.3%	Cross-sectional	N-back task, conflict score of the Attention Network Test	Whole blood	Broad screening of miRNAs expression levels quantified with the SurePrint Human miRNA Microarray rel. 21 (Agilent Technologies, USA) in two rounds of 1126 and 216 samples.	The relationship between miRNA profiles and clinical outcomes was assessed with linear regression models using the limma R package, adjusting by sex and cohort. Multiple testing was corrected with Benjamini–Hochberg method.
Gonzalez-Giraldo [91]	Colombia	Healthy	230	21.2	Participants were university students	Cross-sectional	Stroop test	Whole blood	Genotypification for a functional VNTR in MIR137 gene using PCR.	The association between VNTR genotypes of MIR137 and EF was assessed with a linear regression model, adjusting by age, gender and hours of sleep, using the SNPStats program.
Gou [90]	China	First episode patients with schizophrenia (FEPS)	173 (123 cases, 50 HC)	28.4 (FEPS), 29.1 (HC)	12.9 (FEPS), 14.1 (HC)	Case-controls	Working memory domain of the MCCB	Whole blood	miRNA was detected by reverse transcription and qRT-PCR using synthetic ath-miR-159a as internal control for normalization.	General linear regression analysis evaluated relationships between biological indicators and cognitive scores, adjusted by age, gender, and education year (SPSS).
Han [89]	China	Parkinson´s Disease	138 (PD-NC:39; PD-MCI:37; PDD:22; and 40 HC)	63.8 (HC), 61.5 (PD-NC), 61.4 (PD-MCI), 61.9 (PDD)	12.28 (PD-NC), 8.34 (PD-MCI), 9.55 (PDD)	Case-controls	Stroop test and TMT-B	Serum	The microRNA family was detected using qRT-PCR. Serum miRNAs were extracted using a miRNeasy Serum/Plasma Kit (Qiagen, Germany).	Multivariate linear regression model, after controlling for years of education and UPDRS-III sub-score.
Ignacio [88]	USA	Alcohol use disorders	30 (20 cases, 10 HC)	30.9	NR	Case-controls	Selected scales from the Delis-Kaplan Executive Function System	Serum	Next-generation sequencing (NGS), small RNA libraries were made with the TruSeq Small RNA Sample Prep kit (Illumina), using a MiSeq Benchtop Sequencer (Illumina).	Pearson correlations were calculated. Significance was obtained using an R to T transformation and adjusted for multiple comparisons with Benjamini-Hochberg False Discovery Rate.
Islam [40]	Germany	MCI	132	25.9	NR	Cross-sectional	EF domain: TMT-A and -B, and digit symbol test. WM domain: digit span forwards and backwards.	Whole blood	NGS, TruSeq® Small RNA kit according to manufacturer´s protocol (Illumina, San Diego, CA, USA)	Highly correlated microRNAs were identified and summarized using weighted gene co-expression network analysis (WGCNA) in R (version 1.61).
Liu [87]	China	Healthy	290	22.7	15.5 (TT), 15.7 (TG)	Cross-sectional	Letter 2-back task	Whole blood	MIR137 rs1625579 wasgenotyped using the iPLEX Gold Assay. The accuracy of genotyping was assessed using duplicate analysis.	Two-sample t-tests were used to check for differences in age, education levels, and intelligence quotient scores between the two genotype groups.
Ma [86]	China	Schizophrenia	1239 (611 cases vs 628 HC)	35.6 (cases), 36.5 (HC)	NR	Case-controls	Digit Sequencing Task	Whole blood	The SNP was genotyped using the SNaPshot technique with polymerase chain reaction.	The comparisons of allelic and genotypic distributions were performed using Pearson’s chi squared test or Student’s t-test. Generalized ORs with 95% CIs of the alleles were calculated.
O’Meara [85]	USA	HIV	36 (31 HIV, 5 HC)	45 (higher performing), 51 (lower performing)	16 (higher performing), 13 (lower performing)	Case-controls	TMT-A and -B, Controlled Oral Word Association	Plasma	Next generation sequencing of exosomal microRNA.	Discrimination ability of the microRNA was tested with principal component analysis (R v3.5.0) and receiver operating characteristic curves (ROC, in SAS v9.4).
Pan [84]	China	FEPS	165 (118 cases, 47 HC)	28.7 (cases), 29.6 (HC)	12.6 (cases), 14.1 (HC)	Case-controls	Executive function domain of MCCB	Whole blood	MicroRNA expression was detected using qRT-PCR.	Mann–Whitney U test or independent sample t-test was used to examine differences in miRNA, mRNA, protein expression levels, and EF. Partial correlations adjusting by age, sex, and education were performed.
Park [83]	Republic of Korea	Aging	29 (14 SCA, 15 NCA)	75.7	8	Cross-section	The Frontal Assessment Battery	Buffy coat samples from peripheral blood	DNA extraction and methylation microarray analysis performed using Illumina Infinium Methylation EPIC Bead Chip kits (Illumina, Inc., San Diego, CA, USA).	The differences between groups were tested with the χ2 test, independent t-test, and Mann-Whitey U test. Statistical analyses were performed using the SPSS software v.25.0.
Potkin [82]	USA	Schizophrenia	162 (88 cases, 74 HC)	NR	NR	Cross-section	Sternberg Item Recognition Paradigm	NR	MIR137 genotypification. The potential experimental and predicted microRNA targets were searched in an open access source.	Gene set enrichment analysis using an open access tool.
Qi [81]	China	MDD	204 (81 cases, 123 HC)	29 (cases), 27 (HC)	13.5 (cases), 14.6 (HC)	Case-controls	EF domain of the CANTAB	Whole blood	The microRNA levels were detected using qPCR, using U6 to normalize the values	Two-sample t-tests on mixing coefficients of each multimodal neuroimaging component, to find the joint independent components correlated with microRNA levels.
Schroeder [80]	Germany	Aging	1318	66.76	NR	Cross-sectional	T-score of spatial working memory combined with Wisconsin Card Sorting Test	Whole blood	Genotyping using microarray-based SNP genotyping “Genome-Wide Human SNP Array 6.0” (Affymetrix, Inc.).	In silico prediction of mRNA-microRNA binding based on SNPs results.
Sun [79]	China	MDD	102 (48 cases, 54 HC)	40.5 (cases), 38 (HC)	12 (cases), 15.2 (HC)	Case-controls	Trail Making Test	Blood samples	The microRNA levels were analyzed using qPCR, with normalization with U6.	Correlation analyses were conducted using Pearson’s product-moment correlation coefficient.
Toyama [78]	Japan	MCI	178	73*	NR	Cross-sectional (from ongoing cohort)	EF domain of Montreal Cognitive Assessment (MoCA-J)	Exosomes from serum	Serum exosome microRNA expression levels measured by qPCR methods, TaqMan Assay Kits (Applied Biosystems) with cel-miR-39 for normalization.	Univariate and multivariate logistic regression analyses assessed serum exosome expression and MCI, adjusted by baseline parameters, risk factors, and medications.
van Erp [77]	USA	Schizophrenia	111 (48 cases and 63 HC)	37.0 (cases), 37.6 (HC)	13.3 (cases), 16 (HC)	Case-controls	Sternberg Item Recognition Paradigm	NR	Genotyping of SNP using real-time PCR with TaqMan® probes and primers (Applied Biosystems).	Group (patient/control) and genotype (GG + GT/TT) effects on left DLPFC during a WM task were measured using mixed model regression analyses (SAS 9.2, Proc Mixed).
Wang [76]	China	Cerebral Small Vessel Disease	120 (80 cases and 40 HC)	66.3 (cases)	9.2 (cases)	Case-controls	Executive Function Assessment (EFA)	Exosomes from plasma	High throughput sequencing. Then the expression of the candidate microRNA was validated with qRT-PCR.	Correlation analysis performed with Spearman’s rank correlation coefficient.
Zhang [75]	China	MCI in T2DM	117 [80 controls (no MCI), and 37 with MCI, all T2DM]	59.0 (cases), 53.5 (HC)	12 (MCI), 12 (HC)	Case-controls	Digit Span Test, Verbal Fluency Test, and TMT-B	Exosomes from Plasma	The expression of exosomal microRNAs was detected with qPCR and normalized to U6.	The association between miR-let-7c-5p and MCI, was assessed using Pearson and partial correlation analyses, as well as binary logistic analyses, and multiple linear regression.

*ADHD* attention-deficit/hyperactivity disorder, *CANTAB* cambridge neuropsychological test automated battery, *EF* executive functions, *HIV* human immunodeficiency virus, *HC* healthy controls, *MCCB* measurement and treatment research to improve cognition in schizophrenia (MATRICS) consensus cognitive battery, *MCI* mild cognitive impairment, *NCA* normal cognitive aging, *NR* not reported, *PCR* polymerase chain reaction, *PD* parkinson´s disease, *PD-NC* PD patients with normal cognition, *PD-MCI* PD with mild cognitive impairment, *PDD* PD and dementia, *RCT* randomized controlled trial, *SCA* successful cognitive aging, *SNPs* single nucleotide polymorphisms, *T2DM* Type 2 diabetes mellitus, *TMT-4* number-letter-sequencing from the delis-kaplan executive function system, *UPDRS-III* unified parkinson’s disease rating scale, part III, *VNTR* variable number tandem repeat, *WM* working memory, *WMS* wechsler memory scale.

^**┼**^ mean age of the participants when available, if not the median of the overall group or the mean/median of each subgroup of participants was included.

*median.

**Table 2:** Characteristics of the animal studies.

Study	Disease model	Animal description	n	Groups	Behavioral test	Measure Timing of the EFs	MicroRNA model	Tissue analyzed/manipulated
Berchenko [115]	*AD:* stereotactic administration of aggregated Aβ40 Human in hippocampus.	Male, 15-16 months rats	33	Experimental group (n = 17), comparison group (n = 16)	Novel object recognition	Baseline, 5–6 days after intrahippocampal Aβ40 aggregates administration, 8–9 days after intranasal miR administration.	Experimental group: Intranasal administration of liposomal miR-101 for 10 days. Control group: No intervention.	Hippocampus
Chu [119]	*Intoxication:* Administration of PS NPs.	Male, 5-week-old, C57BL/6 mice	40	4 groups of 10, based on doses: 0, 10, 25, 50 mg/kg of PS NPs	Eight-arm radial maze	After six months of the beginning of trial.	RNA sequencing in PFCs for microRNAs, for two groups: 0 and 50 mg/kg.	PFC
Diamantopoulou [118]	*22q11.2 deletion:* Df(16)A +/− deficiency and Mirta22/Emc10 loss of function	Male, 8-week-old transgenic mice	60	Df (16)A + /– and Mirta22 + /–: 16; Mirta22 + /–: 14; Df(16)A + /–: 13; and WT: 17	T-maze	At 8 weeks	Transgenic mice	mPFC slice
Fenelon [114]	*22q11.2 deletion:* 1.3-Mb chromosomal deficiency on mouse syntenic chromosome 16, [Df(16)A + /− mice]. And heterozygous Dgcr8 mutant mice	Male, 4-6 weeks old, Dgcr8 +/− mutant and wild type mice	NR	NR	Electrophysiology study	NR	Deletion of the Dgcr8 gene	PFC
Fenelon [113]	*22q11.2 deletion:* Df(16)A +/- mice	Male, adult Df(16)A +/- and wildtype mice	28	[n = 14 WT, n = 14 Df(16)A +/− ]	Novel object recognition	NR	Deletion of the Dgcr8 gene	Brain
Gai [112]	*Hypoxia ischemia in neonates:* ligation (active) or separation (sham) of common carotid artery at PND7.	Neonatal C57BL/6 J	NR	Sham and active. Active included: ligation + negative control, and ligation + miR mimics.	Y maze test	28 days after the HI injury	Stereotactic injection of miR-9-5p mimics or negative control (NC) into the lateral ventricle at PND4.	Brain
Henry [111]	*TBI*: Controlled cortical impact (CCI)	Male, 10- 12 weeks old, C57Bl/6 J mice	>10	Study 1: Sham vs CCI, perfused at 1 h, 24 h, 72 h, and 7 days post-injury. One set for RNA extraction, another only at 7 days for microglia differentiation (n = 6/g). Study 2: CCI mice (n = 5-6/g) plus antagomir or NC injection. Study 3: CCI mice (n = 8/g) with miR-155 antagomir vs NC. Study 4: CCI continuous infusion vs NC, and sham injured as controls. Study 5: CCI mice (n = 12–15/g) plus delayed infusion vs NC	Y maze test	7 days post injury (PID7, “acute”), and PID11 (“chronic”)	*Acute intracerebral ventricular (ICV) administration:* Injection of miR-155 antagomir or negative control (artificial cerebrospinal fluid) into the left lateral ventricle 15 min post injury. *Delayed continuous ICV administration*: Immediately prior to CCI, the right lateral ventricle was stereotaxically perforated. 24 h after injury, the infusion cannula was connected to a mini-osmotic pump implanted behind the scapula. Osmotic pumps were primed for ~8 h prior to implantation and were filled with miR-155 antagomir (0.5 nmol/day) or NC, with continuous infusion for 6 days at a rate of 0.5 μl/h.	Cortex and Hippocampus
Islam [40]	Age-associated memory decline	Male, 12 months-old, C57B/6 J wild‐type mice	66	Discovery sample (n = 10). Validation groups: 3-month-old control (n = 18), 16,5-month-old injected with scrambled (n = 18), and 16,5-month-old mice injected with microRNA inhibitors (n = 20)	Morris water maze	At 13.5, 15, 16.5 months	High-throughput Small RNA sequencing and bioinformatic analysis from the blood and brain regions from mice. Stereotactic injection of anti-miR-mix (candidate microRNAs) vs scramble oligonucleotides.	NGS: Blood, ACC, CA1, CA3, and DG regions. Injection: hippocampal CA region
Kawakita [117]	*AD:* senescence‑accelerated mouse prone 8 (SAMP8), and senescence‑accelerated mouse resistant 1 (SAMR1) mice as controls.	Male, 5-month-old, 30-40 g, SAMR1 and SAMP8 mice.	12	SAMP8 (n = 6/g) and SAMR1 mice (n = 6/g).	Y maze test	5 months of age	RNA was extracted from the tissue (brainstem) using the miRNeasy Mini Kit (Qiagen, Inc.). Micro arrays were ensembled and expression levels of miRNA were measured with qPCR normalized to sno‑RNA‑202.	Brainstem
Koh [110]	*AD:* 5XFAD	8 months old, B6SJLF1/J (JAX#100012) and 5XFAD transgenic mice	10–14	control (n = 5-7) or miR-485-3p ASO (n = 5-7)	Y maze test	Not reported	Intracerebroventricular (ICV) stereotactic injection, of both the miR-485-3p antisense oligonucleotide samples and the NC at 8- or 10-months of age once weekly for 2 weeks.	Brain
Liu [116]	*AD:* infusion of Aβ1-42	Male, Sprague-Dawley rats (200-250 g)	42	control (n = 10); AD (n = 12); AD infused with and miR-155 inhibitor (n = 12); AD + miR155 inhibitor scramble (n = 8); and AD and individual PIC receptor inhibitor	Y maze	Two and four weeks after infusion of Ab1-42	Using intracerebroventricular canulation, miR-155 inhibitor was injected as continuous infusion.	Parietal cortex, hippocampus and amygdala.
Liu [109]	Age-related cognitive decline	Aged rats, and genetically modified mice	Not reported	Not reported	Delayed Matching-to-Place (DMP) water maze	Not reported	1) miR-124-3 knockout mice: 2) Overexpression model: stereotactic injection of viral vector with miRNA mimic in both hemispheres.	In rats: Hippocampus and parietal cortex. In mice: systemic effects
Malmevik [108]	Inhibition of microRNA in hippocampus	Male, 10-weeks old, C57BL/6 mice	12	For each miRNA experiment (miR-9 and miR-34), three mice injected with AAV-GFP control (GFP Ctrl) and three mice injected with either AAV-GFP-miR-9sp or –miR-34sp were used.	Custom-made closed plus maze	Two weeks after injection	miRNA inhibition: by intra-hippocampal injections of AAV vector suspensions containing miRNA sponges. Transgenic mice: Transgenic miR.34.T.GFP sensor mice using lentiviral transgenesis, and miR-124.T.GFP and miR-9.T.GFP transgenic mice.	Hippocampi
Ouchi [107]	*22q11.2 DS:* transgenic KO Dgcr8 mice	Male and female 2-month-old, Dgcr8 +/− or -/- mice	31	Dgcr8 + /+, n = 10 for males, n = 5 for females; Dgcr8 +/− , n = 10 for males, n = 6 for Females.	Y maze	NR	Deletion of the Dgcr8 genes	Hippocampus
Qiu [106]	*Altered Biogenesis:* DICER ablation	Male, 2 months-old VIP^Cre^, Dicer^f^, and Ai14 mice	Not reported	Not reported	Y maze test	2 months of age	VIP^Cre^, Dicer^f^, and Ai14 transgenic mice as reported previously.	Systemic effects
Sarkar [105]	*Cognitive impairment:* Transgenic miR-34a +/- mice.	miR-34a + /− mice	Not reported	Not reported	T-maze forced alternation and Y-maze	49–65 days of continuous NC or doxy exposure, starting at 4 – 4.5 d	Transgenic conditional (tetracycline) overexpressing miR-34a mice.	Entorhinal cortex, hippocampus, PFC and thalamus
Shan [104]	*Intoxication:* anesthetics-induced dysfunction	Male, 18 months (aged) Wistar rats	60	1.5% isoflurane, 3% sevoflurane, vehicle gas each for 2 and 4 h.	Y-maze test	Four days after stopping anesthesia (day 8)	Aged rats were exposed to isoflurane, and after perfusion, the expression of miR-190a, miR-190b, miR-31, and miR-30a in one-side whole hippocampus and mPFC were measured with qRT-PCR.	Hippocampus and mPFC
Toyama [103]	*Vascular cognitive impairment:* bilateral common carotid artery stenosis (BCAS) surgery	Male, C57BL/6 wild-type mice	19 (1° cohort), 18 (2° cohort)	1^st^ cohort: sham (n = 6); scr-miR–treated BCAS [n = 6]; and anti-miR–treated BCAS (n = 7). 2^nd^ cohort: sham (n = 5); scr-miR–treated BCAS (n = 6); and anti-miR treated BCAS [n = 7]).	Y maze test	at 11 weeks old, and 12 weeks old	Intraperitoneal injection of either LNA-anti-miR-501-3p or scrambled miR as control). The concentrations of anti-miR or scrambled miR included both 1 and 10 mg/kg-body weight. A single injection was performed 1 h after BCAS surgery.	White matter and Hippocampus
Wang [102]	*Intoxication:* Perinatal fluoride exposure	Both genders, pups of ICR mice	15	Pregnant mice receiving: 0, 25, 50, and 100 mg/L NaF until ablactation	8- arm maze	Trial lasted 7 days, PN day of beginning NR.	Expression levels of two microRNAs were measured using RT-qPCR.	Hippocampus
Xu [100]	*Cognitive impairment:* miR-30e over expressing rat.	Male, 4 weeks old, Sprague-Dawley (SD) rats	40	8 in each group: i) Control; ii) lenti-GFP; iii) lenti-miR-30e; iv) lenti-miR-30e plus CG; and v) lenti-miR-30e plus fluoxetine group.	Morris water maze	Rats were 8 weeks old (at the end of drug administration)	Injection of lentivirus containing rat miR-30e into the hippocampal dentate gyrus at a rate of 0.2 µl/min. The rats were allowed to recover for 14 days	Hippocampal dentate gyrus
Xu [101]	*ADHD:* spontaneously hypertensive rat (SHR)	male, 4 weeks old, SHR and Wistar Kyoto (WKY) rats	46	Four groups: miR-OV (n = 11), NC (n = 11), miR-SH (n = 12), NC-SH (n = 12)	Morris water maze	One week after injection of lentivirus (at 5 weeks old)	Lentiviral vectors were injected into the lateral ventricles stereotactically.	Brain, PFC
Zhang [99]	*Induced aging:* Administration of D-galactose	male, 17-week-old male C57BL/6 J mice	60	normal (n = 15) control group, D-gal+PBS (n = 15), D-gal+Cell (n = 15), D-gal+Cell-anti-miR-206 (n = 15)	Y-maze	during a 10-day rest period after treatment	miR-206 inhibitors and NC were transfected into hUCMSCs (50 nmol/L). At P5, they were transferred into 6-well plates to a density of 80–90%, and the suspension was injected in the lateral ventricle.	Hippocampus
Zhu [98]	*HIV:* ecoHIV infected mice	Male 8–12-week-old C57BL/6 J mice	6–8	EcoHIV infected vs controls. In each group two subgroups: PDDC treatment or sham injection.	RAWM	After at least 10 days of daily drug treatment.	Measure of microRNA expression by small RNA sequencing.	PFC and HPC

*/g* per group; *5XFAD* five familial AD mutation (5XFAD), *AAV* adeno-associated viral transfer vectors, *Aβ* amyloid beta, *ACC* anterior cingulate cortex, *AD* alzheimer’s disease, *ADHD* attention-deficit hyperactivity disorder, *ASO* antisense oligonucleotide, *BCAS* bilateral common carotid artery stenosis, *CA* cornu ammonis, *DS* deletion syndrome, *DG* dentate gyrus, *GFP* green fluorescent protein, *HIV* human immunodeficiency virus, *hUCMSCs* human umbilical cord mesenchymal stem cells, *Intox. w*. intoxication with, *miR* microRNA, *(m)PFC* (medial) prefrontal cortex, *n* total sample size, *NaF* sodium fluoride, *NC* negative control, *NR* not reported, *OV* overexpressing, *PDDC* HAS-(1-(3-(3,4-dimethoxyphenyl)-2,6-dimethylimidazo[1,2-b]yrrolidin-8-yl)yrrolidine-3-yl)-carbamate, *PND* post-natal day, *PS NPs* polystyrene nanoplastics, *RAWM* radial arm water maze, *SH* inhibiting vector, *TBI* traumatic brain injury, *WT* wild type.

The included studies evaluated various disruptions along the microRNA biosynthesis process and their subsequent effects on EFs. Some studies used more than one method. Alterations in the genes coding for microRNAs were reported in nine human studies [77, 80, 82, 83, 86, 87, 91, 93, 96] (including gene mutations, variable number tandem repeats [VNTRs], and gene methylation) and in two animal studies (one overexpression [109] and one knockout [KO] transgenic mouse model [105]). The way in which reduced availability of the nuclear enzyme DGCR8 affects working memory was studied in four mice models of the 22q11.2 deletion syndrome (22q11.2 DS) [107, 113, 114, 118]; the manipulation of the cytoplasmic enzyme DICER was studied in a KO mouse model [106]; the expression of microRNAs was manipulated in 11 animal studies [40, 99–101, 103, 108–112, 115, 116] (by injection of mimics or microRNA inhibitors directly into the animal, e.g., by intracerebroventricular injection or peritoneal injection); and the differential expression of microRNAs was studied in 15 human studies [75, 76, 78, 79, 81, 84, 85, 88–90, 92, 94, 95, 97] (14 in blood, and one in saliva) and six animal studies [40, 98, 102, 104, 117, 119] (one in blood, one in the brainstem, three in the hippocampus, and three in the prefrontal cortex). These processes and the related findings are presented in Fig. 1.

**Fig. 2:**
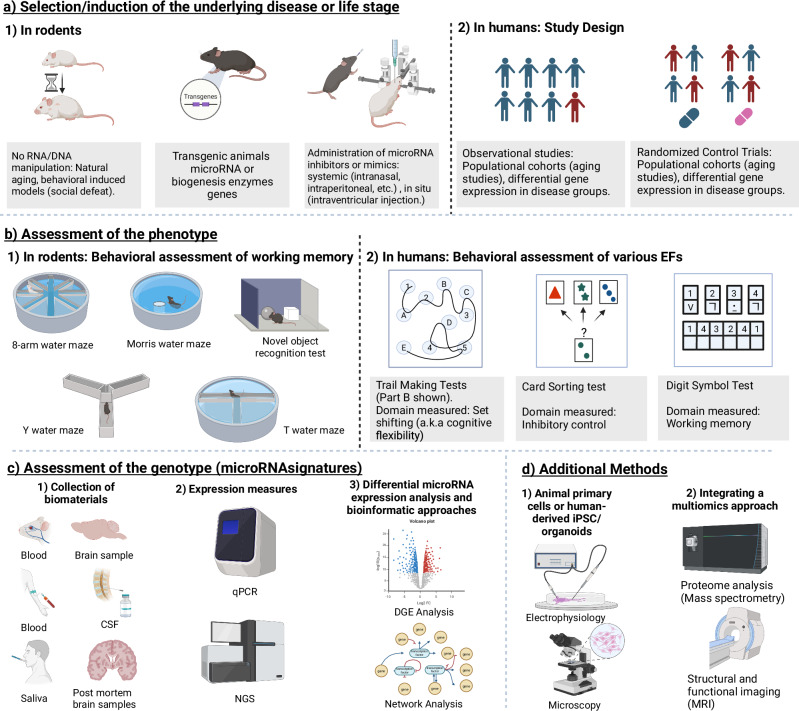
Techniques used for investigating microRNAs and executive functions in human and animal models. The methods used to assess the role of microRNA in executive functions (EFs) differ in various aspects. The most common were: (**a**) Regarding the selection or induction of the underlying pathology or age group being studied: a.1) In animal models: no genetic manipulation (examples are natural aging by using older animals or other behaviorally induced models, such as early life stress or social defeat); use of transgenic animals (with manipulation of genes such as DGCR8 or DICER that code for relevant enzymes related to the biosynthesis process); and administration of microRNAs inhibitors or mimics peripherally, e.g., by intranasal injection, or directly into the brain by stereotactic injections into the interventricular area or hippocampus; and a.2) In human studies: observational cohort, case-control, or cross-sectional studies without an intervention and experimental designs such as randomized controlled trials. **b** Regarding the methods used for assessing the phenotype, i.e., executive functioning: b.1) In animal models: behavioral assessments of working memory (other domains are still barely established) with the eight-arm water maze, Morris water maze, Novel Object Recognition test, Y water maze, or T water maze (these evaluations may require correction for visual and motor abilities); and b.2) In human studies: the domains of executive functions are broader, for instance the Trail Making Test can measure set shifting, the Card Sorting Test evaluates inhibitory control, and the digit symbol test assesses working memory. 3) Regarding the assessment of the genotype or the microRNA changes: 3a) sample collection, with examples of biomaterial ranging from saliva to brain-specific areas collected post mortem; 3b) measurement of microRNA expression either with tests for specific microRNAs that use polymerase chain reaction methods or with broader analysis by sequencing techniques; and (**c**) differential microRNA expression analysis by using pipelines in programming or statistical software, with possible further bioinformatics analysis, e.g., network analysis or gene set enrichment analysis. And (**d**) Regarding additional methods that can complement the research, for example: d.1) studies of cellular electrical or morphological changes after microRNA manipulation (with inhibitors or mimics) in cellular cultures derived from primary or human-derived induced pluripotent stem cells or organoids; and d.2) other omics techniques, such as proteome analysis or correlations with changes in structural or functional imaging markers. Created in BioRender. Navarro Flores, A. (2026) https://BioRender.com/ztbg5cd. Partially adapted from Faber, S. [164].

Various animal models were included, and the models also used genetic engineering, electrophysiological measures, and immunohistochemistry methods. The most frequently used methods of analysis were GWASs (humans), and quantitative polymerase chain reaction (qPCR) and next-generation sequencing (both humans and animals). A summary of the common pipelines is presented in Fig. 2.

The disease models most commonly evaluated in the animal studies were cognitive impairment secondary to various injuries (n = 8) [100, 102–105, 111, 112, 119], Alzheimer disease (n = 4) [110, 115–117], 22q11.2 deletion syndrome (n = 4) [107, 113, 114, 118], and age-related mild cognitive impairment (MCI, n = 3) [40, 99, 109]. In the human studies, the conditions studied were both primary MCI and MCI secondary to other chronic conditions (n = 9) [40, 75, 76, 78, 85, 89, 94, 95, 97], schizophrenia (n = 6) [77, 82, 84, 86, 90, 93], cognitive aging (n = 3) [80, 83, 96], major depressive disorder (n = 2) [79, 81], and normal variations of cognition in healthy individuals (n = 2) [87, 91].

#### Quality assessment

In the human cohorts, items 2 (selection of the non-exposed cohort), 3 (ascertainment of the exposure), and 5 (comparability of cohorts) had a low mean risk of bias and items 1 (representativeness of the sample), 6 (outcome assessment), and 8 (follow-up), had a high mean risk of bias. In the case-control studies, items 1 (sample representativeness) and 4 (validated tool) had a low mean risk of bias; items 5 (comparability of samples) and 6 (outcome assessment) had a moderate mean risk of bias; and items 2 (sample size), 3 (characteristics of the non-respondents), and 7 (statistical test) had a high mean risk of bias. The only randomized controlled trial (RCT) included had a high risk of bias [97].

In animal studies, a low to moderate risk of bias was found only for the following items: similar baseline characteristics between groups, missing data, and other sources of bias, in this case financing from industry agencies. The rest of the items presented a moderate to high risk of bias.

A detailed depiction of the risk of bias assessment is presented in the Supplement (eTables 8–9, eFigures 2–4).

### Changes in microRNA-related genes

#### MIR137 variants: the most studied microRNA gene mutation impairs EFs

Gonzalez-Tirado et al. [91] performed a study in 230 healthy young Colombian adults (mean age, 21.2 years) and used polymerase chain reaction (PCR) to genotype the sample according to a functional VNTR for the MIR137 gene. They found that individuals who carried one copy of the 4-repeat allele showed better executive functioning, as measured by the Stroop test (Stroop facilitation scores). Liu et al. [87] performed a cross-sectional study in healthy participants in China (N = 290) and genotyped them for MIR137 rs1625579. On the basis of a functional magnetic resonance imaging experiment, they concluded that this single-nucleotide polymorphism (SNP) was associated with reduced connectivity in the dorsolateral prefrontal cortex and reduced working memory performance. Similarly, Ma et al. [86] conducted a case-control study of Chinese adults with schizophrenia (N = 1239; 611 cases and 628 controls) and evaluated working memory with the Digit Sequencing Task. They found that the T allele for the same SNP (rs1625579) was associated with lower working memory scores. Cosgrove et al. [93] performed a case-control study of 1000 Irish adults (808 patients with schizophrenia, 192 controls), most of them male (99%). The group studied the polygenic risk of the affected downstream genes that were targets of or were regulated by miR-137 and were relevant in cognition (including EFs) and found that the miR-137 polygenic score was inversely correlated with intelligence quotient, attention, and memory (i.e., episodic, visual, and working memory). Potkin et al. [82] combined data on gene differences identified in a GWAS with data from a working memory paradigm performed during functional magnetic resonance imaging [82]. The candidate genes found in the GWAS were submitted to an enrichment network analysis by using a gene set collection of experimental and putative targets, including microRNAs, curated by the authors. The authors found that the compromised genes regulated the expression of microRNA genes, including miR137, miR-448, and miR-218.

Negative results have also been reported for the MIR137 gene: Van Erp et al. [77] conducted a small case-control study in the USA in patients with schizophrenia (N = 111; 48 cases and 63 controls) and evaluated working memory with the Sternberg Item Recognition Paradigm during functional neuroimaging. They found higher hyperactivation of the dorsolateral prefrontal cortex and lower working memory scores in the patients than in the controls but no genotype-phenotype association.

#### Mutations in other genes related to microRNAs

SNPs in the MIR2113 gene were suggested to be related to general cognitive function. Andrews et al. studied this genetic marker in association with longitudinal cognitive performance, including working memory, across 12 years in a population of cognitively healthy older adults. They did not find an association with working memory, but they did find that the marker was related to an accelerated decline in episodic memory [96]. Similarly, Schroeder et al. studied working memory in older adults by using GWAS data. [80] The most significant SNP related to working memory performance was rs113948889, but additional SNPs were found to be related to other cognitive functions. The authors used a bioinformatic tool to predict which other proxy SNPs (in linkage disequilibrium with the ones found in their GWAS) were associated with microRNA-mRNA binding and found that rs1044950 (in linkage disequilibrium with rs113948889) was predicted to interfere with the binding of hsa-miR-138-5p and DCP1B. This relationship was confirmed with the luciferase assay in HEK293 cells. In postmortem human brains, both hsa-miR-138-5p and DCP1B were found in the areas of the hippocampus and frontal cortex, suggesting that an interaction may occur.

#### Effects of gene methylation on EFs

Some individuals maintain above average performance even as they age. Park et al. [83] performed a study in Korean older adults with above average performance in all cognitive domains, including EFs. They performed a methylation assay that included a microRNA gene site named *MIR885* and found differentially methylated CpGs in the promoter regions of the *MIR885* gene. The relative differentially methylated genes expression of this gene was two-fold higher in the group with successful cognitive aging than in the group with normal cognitive aging.

### Reduced availability of nuclear microRNA processing enzymes

DiGeorge critical region 8 (DGCR8) is a subunit of the microprocessor complex that is required for the maturation of the longer primary microRNA into its short precursor form. [120] In 22q11.2 deletion syndrome, a neurodevelopmental disorder, both the DGCR8 and also other microRNA genes could be affected depending on the size of the deletion; consequently, an expression dysregulation is observed across the microRNAome. The phenotype includes cognitive impairment; a high risk for schizophrenia and other psychiatric disorders, such as anxiety and depression; and multiple physical malformations [121]. Fenelon et al. [114] engineered a mouse model of 22q11.2 DS by using a syntenic mutation. The DF16 +/− mouse was heterozygous to this mutation, and in the pyramidal neurons of the prefrontal cortex of this mouse, the authors found abnormal morphology that—during brief stimulation trains—was associated with short-term synaptic depression and a reduced initial phase of synaptic potentiation. These alterations in synaptic plasticity could explain the cognitive deficits in 22q11.2 DS and may provide a link between microRNA expression and higher order functions. In a later study, Fenelon et al. [113] found that mice with this mutation performed poorly in the Novel Object Recognition test of working memory. Another behavioral experiment was performed by Ouchi et al. [107] in a transgenic KO Dgcr8 mouse model: The group evaluated working memory with the Morris water maze and the Y maze and found reduced performance in these hippocampus-dependent tasks. The reduction in performance correlated with schizophrenia-related genes that were downregulated in this brain area and with a reduction in adult hippocampal stem/progenitor cells. Working memory performance improved after administration of insulin-like growth factor 2 in the hippocampus, a previously reported candidate for this function that was severely decreased in the mouse model [107].

Diamantopoulou et al. [118] also used the DF16 +/−  mouse model along with a loss of function mutation in a gene with a major transcriptional effect due to microRNA dysregulation, Mirta22/Emc10. By adding this mutation, the authors rescued the schizophrenia-related phenotype of the mice, including impaired working memory performance [118].

### Reduced availability of cytoplasmic microRNA processing enzymes

DICER is a cytoplasmic enzyme responsible for cleaving a microRNA into its mature form. Qiu et al. [106] generated a mouse model of DICER KO and found that postmitotic ablation of DICER in cortical vasoactive intestinal peptide-expressing interneurons (which are relevant for executive control) produced progressive cell loss in adulthood [106]. These mutant mice presented with a shortened life span and, paradoxically, performed better in the spatial working memory paradigm, even though learning and memory were not enhanced. Administration of valproate incremented the life span and reduced performance to levels similar to those in controls. The authors hypothesized that GABAergic circuits could be enhanced in and be responsible for this phenotype.

### Differential microRNA expression correlates with EFs

Studies reported dysregulation of microRNAs in both directions, i.e., upregulated and downregulated, and an association of microRNA dysregulation with increased and decreased EFs. A summary of the microRNA findings reported in at least two samples is presented below, and the full list of dysregulated microRNAs can be found in Table 3. Moreover, based on a recent report of microRNA expression in the human prefrontal cortex, [53] the reads per million of each candidate were included in this table to complement the interpretation of the results.

**Table 3:** Description of the microRNAs reported as significantly dysregulated in the literature.

microRNA	dlPFC expression (mean RPM)*	Regulation	EFs	EFs test	Disorder	Source of miRNA information	Specifications	Humans	Animals
miR-101	17,310 (3p) 7.8 (5p)	up	increased	Novel object recognition	Alzheimer’s Disease	Injected in the nose	-	-	Berchenko [115]
miR-1207-5p	NM	down	increased	Digit Span Backward	HIV	Peripheral blood	Plasma	Aparicio [95]	-
miR-124-3p	5035.2	up	declined	8-arm maze test	Intoxication with fluoride	Brain	Hippocampus	-	Wang [102]
		down	declined	DMP water maze	Age-related MCI	Genetic modification	-	-	Liu [109]
		down	increased	Plus maze	Selected miRNA inhibition	Brain	Intrahippocampal inhibition	-	Malmevik [108]
miR-126-3p	29,702.2	up	declined	Radial arm maze	Intoxication with plastic particles	Brain	PFC	-	Chu [119]
hsa-mir-1260b-3p	3.9 (single isoform)	down	declined	Radial arm maze	Intoxication with plastic particles	Brain	PFC	-	Chu [119]
hsa-mir-1260b-5p	3.9 (single isoform)	down	declined	Radial arm maze	Intoxication with plastic particles	Brain	PFC	-	Chu [119]
miR-1282	NM	down	increased	Digit Span Backward	HIV	Peripheral blood	Plasma	Aparicio [95]	-
miR-132	875.0 (3p) 625.4 (5p)	up	declined	Humans: Intra-Extra Dimensional Set Shift Rodents: 8-arm maze test	Humans: MDD Rodents: Intoxication with fluoride	Humans: peripheral blood Rodents: Brain	Humans: Whole blood, Rodents: Hippocampus	Qi [81]	Wang [102]
miR-133a-5p	2.6	up	declined	Radial arm maze	Intoxication with plastic particles	Brain	PFC	-	Chu [119]
miR-139-5p	5939.2	down	declined	Radial arm maze	Intoxication with plastic particles	Brain	PFC	-	Chu [119]
miR-146a-5p	921.5	up	declined	Humans: Digit span (forward and backward) for working memory; digit symbol test, trail making tests for the executive function domain. Rodents: Morris water maze	MCI	Peripheral blood	Blood	Islam [40]	Islam [40]
miR-148a-3p	4412.9	up	declined	Humans: Digit span (forward and backward) for working memory; digit symbol test, trail making tests for the executive function domain (Islam 2021). Trail making tests, Controlled Oral Word Association (O’Meara 2019). Rodents: Morris water maze.	MCI (Islam 2021), HIV (O’Meara 2019)	Peripheral blood	Blood	Islam [40], O’Meara [85]	Islam [40]
miR-152-3p	835.9	up	declined	Radial arm maze	Intoxication with plastic particles	Brain	PFC	-	Chu [119]
miR-155	88.3 (5p)	down	increased	Y-maze (Henry 2019), Morris Water Maze (Liu 2019)	TBI (Henry 2019), Alzheimer’s Disease (Liu 2019)	Brain	Cortex and hippocampus (Henry [111]), hippocampus (Liu [116])	-	Henry [111], Liu [116]
miR-16-5p	1318.3	up	increased	Digit Span Backward	HIV	Peripheral blood	Plasma	Aparicio [95]	-
		up	declined	Radial arm maze	Intoxication with plastic particles	Brain	PFC	-	Chu [119]
miR-181a-5p	20,501.4	up	declined	Humans: Digit span (forward and backward) for working memory; digit symbol test, trail making tests for the executive function domain. Rodents: Morris water maze	Mild cognitive impairment	Peripheral blood	Blood	Islam [40]	Islam [40]
miR-181b-5p	3966.1	up	declined	MCCB	Schizophrenia	Peripheral blood	Plasma	Gou [90]	-
miR-183-5p	25.0	up	declined	Radial arm water maze	HIV	Brain	PFC and Hippocampus	-	Zhu [98]
miR-190a	1.8 (3p) 55.9 (5p)	down	declined	Y-maze	Intoxication with anesthetics	Brain	Hippocampus and mPFC	-	Shan [104]
miR-190b	1.4 (5p)	down	declined	Y-maze	Intoxication with anesthetics	Brain	Hippocampus and mPFC	-	Shan [104]
miR-190b-5p	1.4	up	increased	Digit Span Backward	HIV	Peripheral blood	Plasma	Aparicio [95]	-
miR-1910-5p	NM	down	increased	Digit Span Backward	HIV	Peripheral blood	Plasma	Aparicio [95]	-
miR-195	29.0 (3p) 650.3 (5p)	either	non-significant	MCCB total score and seven domains	Schizophrenia	Peripheral blood	Blood	Pan [84]	-
mmu-miR-199a-5p	217.6	up	declined	Radial arm maze	Intoxication with plastic particles	Brain	PFC	-	Chu [119]
mmu-miR-199b-3p	608.9	up	declined	Radial arm maze	Intoxication with plastic particles	Brain	PFC	-	Chu [119]
mmu-miR-199b-5p	80.5	up	declined	Radial arm maze	Intoxication with plastic particles	Brain	PFC	-	Chu [119]
miR-200b-3p	38.6	up	declined	RAWM	HIV	Brain	PFC and Hippocampus	-	Zhu [98]
miR-200c-3p	10.7	up	declined	RAWM	HIV	Brain	PFC and Hippocampus	-	Zhu [98]
		up	increased	Digit Span Backward	HIV	Peripheral blood	Plasma	Aparicio [95]	-
miR-206	2.9	down	increased	Y-maze	Induced aging	Intraventricular injection	Hippocampus analyzed	-	Zhang [99]
miR-216b-3p	NM	up	declined	Trail Making tests, Controlled Oral Word Association	HIV	Peripheral blood	Plasma	O'Meara [85]	-
miR-216b-5p	2.2	down	declined	Radial arm maze	Intoxication with plastic particles	Brain	PFC	-	Chu [119]
miR-2214	NM	up	increased	Digit Span Backward	HIV	Peripheral blood	Plasma	Aparicio [95]	-
miR-222-3p	NM	down	declined	Radial arm maze	Intoxication with plastic particles	Brain	PFC	-	Chu [119]
miR-27a	2729.1 (3p) 2.2 (5p)	down	non-significant	Trail Making tests, and the Stroop Color-Word Test	End-stage renal disease	Peripheral blood	Plasma	Bijkerk [94]	-
mmu-miR-28a-3p	324.2	up	declined	Radial arm maze	Intoxication with plastic particles	Brain	PFC	-	Chu [119]
miR-29b	2050.4 (3p) 2.2 (1-5p) 50.9 (2-5p)	up	increased	Stroop Color-Word Test and Trail Making Test B	Parkinson	Peripheral blood	Serum	Han [89]	-
miR-30a	604.9 (3p) 12,531.5 (5p)	down	declined	Y-maze	Intoxication with anesthetics	Brain	Hippocampus and mPFC	-	Shan [104]
miR-30e	793.6 (3p) 3623.6 (5p)	up	declined	Radial arm maze (Chu 2022), Morris water maze (Xu [100])	Intoxication with plastic particles (Chu 2015), selected miRNA overexpression (Xu [100])	Brain	PFC (Chu 2022), Brain (Xu [100])	-	Chu [119], Xu [100]
miR-31	4.5 (3p) 92 (5p)	down	declined	Y-maze	Intoxication with anesthetics	Brain	Hippocampus and mPFC	-	Shan [104]
miR-31-5p	92	up	increased	Digit Span Backward	HIV	Peripheral blood	Plasma	Aparicio [95]	-
miR-3138	NM	up	increased	Digit Span Backward	HIV	Peripheral blood	Plasma	Aparicio [95]	-
miR-3201	NM	down	increased	Digit Span Backward	HIV	Peripheral blood	Plasma	Aparicio [95]	-
miR-320e	NM	up	increased	Executive function assessment	Cerebral Small Vessel Disease	Peripheral blood	Blood	Wang [76]	-
miR-326	76.7	down	non-significant	Trail Making tests, and the Stroop Colour Word Test	End-stage renal disease	Peripheral blood	Plasma	Bijkerk [94]	-
miR-34a	1.9 (3p) 529.2 (5p)	up	declined	T-maze/Y-maze	Selected miRNA overexpression	Genetic modification	Hippocampus and prefrontal cortex analyzed	-	Sarkar [105]
miR-34c-5p	220.0	down	non-significant	Plus maze	Selected miRNA inhibition	Brain	Intrahippocampal inhibition	-	Malmevik [108]
		up	declined	Trail Making tests	MDD	Peripheral blood	Blood samples	Sun [79]	-
mmu-miR-369-5p	154.5	up	declined	Radial arm maze	Intoxication with plastic particles	Brain	PFC	-	Chu [119]
miR-374b-5p	NM	up	increased	Digit Span Backward	HIV	Peripheral blood	Plasma	Aparicio [95]	-
mmu-miR-383-3p	NM	up	declined	Radial arm maze	Intoxication with plastic particles	Brain	PFC	-	Chu [119]
miR-384-5p	NM	up	increased	Morris water maze	ADHD	Intraventricular injection	PFC analyzed	-	Xu [101]
		down	declined	Morris water maze	ADHD	Intraventricular injection	PFC analyzed	-	Xu [101]
miR-3917	NM	down	increased	Digit Span Backward	HIV	Peripheral blood	Plasma	Aparicio [95]	-
miR-4279	NM	up	increased	Digit Span Backward	HIV	Peripheral blood	Plasma	Aparicio [95]	-
miR-429-3p	8.9 (single isoform)	up	declined	Radial arm water maze	HIV	Brain	PFC and Hippocampus	-	Zhu [98]
miR-4423-3p	NM	down	increased	Digit Span Backward	HIV	Peripheral blood	Plasma	Aparicio [95]	-
miR-4734	NM	down	increased	Digit Span Backward	HIV	Peripheral blood	Plasma	Aparicio [95]	-
miR-485-3p	227.0	down	increased	Y-maze	Alzheimer’s Disease	Intraventricular injection	-	-	Koh [110]
miR-501-3p	NM	down	increased	Y-maze	vascular cognitive impairment	Intraperitoneal injection	-	-	Toyama [103]
		up	declined	MOCA executive function domain	Mild cognitive impairment	Peripheral blood	Blood	Toyama [78]	-
miR-504–5p	NM	up	declined	Trail Making tests, Controlled Oral Word Association	HIV	Peripheral blood	Plasma	O'Meara [85]	-
miR-518e-3p	NM	down	increased	Digit Span Backward	HIV	Peripheral blood	Plasma	Aparicio [95]	-
miR-520a-5p	NM	down	increased	Digit Span Backward	HIV	Peripheral blood	Plasma	Aparicio [95]	-
mmu-miR-551b-3p	7.6	up	declined	Radial arm maze	Intoxication with plastic particles	Brain	PFC	-	Chu [119]
miR-652-3p	NM	up	increased	Digit Span Backward	HIV	Peripheral blood	Plasma	Aparicio [95]	-
miR-663a	NM	down	increased	Digit Span Backward	HIV	Peripheral blood	Plasma	Aparicio [95]	-
mmu-miR-708-5p	622.5	up	declined	Radial arm maze	Intoxication with plastic particles	Brain	PFC	-	Chu [119]
miR-9	6820 (3p) 144,593.6 (5p)	up	increased	Ratio scores for Digits, Color trails, Stroop	Mild cognitive impairment	Saliva	Exosomes	Anderson-Hanley [97]	-
miR-9-5p	144,593.6	down	non-significant	Plus maze	Selected miRNA inhibition	Brain	Intrahippocampal inhibition	-	Malmevik [108]
		up	increased	Y-maze	Hipoxia-ischemia	Brain	Cortex	-	Gai [112]
mmu-miR-98-5p	NM	up	declined	Radial arm maze	Intoxication with plastic particles	Brain	PFC	-	Chu [119]
miR-let-7c-5p	6426.2	up	declined	Digit Span Test, Verbal Fluency Test, and Trail Making Test-B	MCI in T2DM	Peripheral blood	Plasma	Zhang [75]	-
miR-let-7f-5p	34,639.5	up	declined	Radial arm maze	Intoxication with plastic particles	Brain	PFC	-	Chu [119]
miR-let-7g-5p	33,529.9	up	declined	Radial arm maze	Intoxication with plastic particles	Brain	PFC	-	Chu [119]
miR-let-7i	22.1 (3p) 16,512.4 (5p)	up	increased	Digit Span Backward	HIV	Peripheral blood	Plasma	Aparicio [95]	-
miR-let-7i-5p	16,512.4	up	declined	Radial arm maze	Intoxication with plastic particles	Brain	PFC	-	Chu [119]

*ADHD* attention-deficit/hyperactivity disorder, *dlPFC* dorsolateral PFC, *DMP* delayed matching-to-place, *HIV* human immunodeficiency virus, *MCI* mild cognitive impairment, *MCCB* measurement and treatment research to improve cognition in schizophrenia (MATRICS) consensus cognitive battery (MCCB), *MDD* major depressive disorder, *MOCA* montreal cognitive assessment, *(m)PFC* (medial) pre-frontal cortex, *NM* not mapped, *T2DM* Type 2 diabetes mellitus, *TBI* traumatic brain injury.

*Mean expression based on dlPFC miRNAs mapped to MiRGeneDB, extracted from Vattathil et al. [53].

#### MicroRNAs consistently differentially expressed within sample types

In humans, miR-148a-3p was found to be upregulated and associated with reduced EFs in two different studies. [40, 85] Islam et al. [40] analyzed neurotypical participants from the PsyCourse Study [122] by assessing EFs with the Trail Making Test and Digit Symbol Test and working memory with the Digit Span Test. The authors performed a microRNAome sequencing analysis with total blood from these individuals and found that miR-148a-3p was significantly increased in participants who had MCI and were therefore at risk of developing dementia. In the other study, O’Meara et al. [85] measured EF performance in 31 patients with HIV who were at risk of cognitive decline. EFs were evaluated with the Trail Making Test parts A and B and the Controlled Oral Word Association Test. MicroRNA expression was measured by next-generation sequencing of plasma exosomes and compared between HIV patients with lower and higher EF performance, and the study found that miR-148a-3p was significantly upregulated in the former group.

In rodents, we found three microRNAs to be consistently dysregulated across samples: miR-155, miR-30e, and miR-384-5p. In a mouse model of traumatic brain injury, miR-155 was highly expressed in the microglia/microphages of the injured cortex. After injection of the inhibitor miR-155 antagomir and the consequent downregulation of miR-155 in the hippocampus, hippocampal expression of the pro-inflammatory biomarkers ITGAM, CD68, NOX2, p22phox, tumor necrosis factor alpha, and CCL2 was significantly reduced and the impairment of working memory was attenuated [111]. Similarly, inhibition of this microRNA in rats was associated with reduced expression of pro-inflammatory genes and rescued the impaired cognitive phenotype [116].

Xu et al. [100] found that rats overexpressing miRNA-30e in the central nervous system had cognitive impairments as measured with the Morris water maze. [100] Similarly, Chu et al. [119] showed in a mouse model of intoxication by polystyrene nano-plastics that upregulation of miRNA-30e was associated with reduced performance in the radial arm maze. [119] Finally, in a rat model of attention-deficit/hyperactivity disorder Xu et al. [101] found in two different samples that overexpression of miR-384—a highly conserved microRNA in humans and rodents—was associated with increased performance in the Morris water maze, whereas, in a different sample of rats, downregulation was associated with decreased performance in the same behavioral test. [101]

#### MicroRNAs consistently differentially expressed across sample types

The following microRNAs were reported as having functional consistency in at least one human and one animal sample: miR-132, miR-146a-5p, miR-148-3p, miR-181a-5p, miR-190b, miR-31, miR-501-3p, and miR-9-5p.

A study by Islam et al. [40] found that in addition to miE-148a-3p, the microRNAs miR-181a-5p and miR-146a-5p were also upregulated and associated with declined EFs in healthy human controls and mice models. In this study, mimics of the three microRNAs were injected into primary cultures of hippocampal neurons and immortalized microglia showing upregulation of genes related to neuronal plasticity, which is required for learning, and memory. Inhibitors of these microRNAs injected into the hippocampus of aged mice rescued the working memory performance up to levels comparable to those in young mice, indicating that these microRNAs may be a potential therapeutic option for MCI.

Qi et al. [81] assessed the effects on EFs of miR-132 expression in medication-naïve Chinese patients with depression. In these patients, miR-132 expression was upregulated and associated with reduced activation in the medial prefrontal cortex, an area relevant for executive performance. The authors found that grey matter thinning in prefrontal and hippocampal areas was associated with reduced scores in the EF test (Intra-Extra Dimensional Set Shift). This microRNA was also studied by a group examining the toxic effects on cognition produced by intrauterine fluoride exposure in mice [102]. The number of errors in the eight-arm maze test was higher in the exposed animals and in the highest dose group, and the hippocampal levels of miR-132 were higher in the exposed than in the non-exposed group. The levels of miR-124 and the DGCR8 gene were also incrementally increased [102].

In a sample of patients living with AIDS, miR-31-5p and miR-190b-5p were positively correlated with the scores on EF tests [95]. In line with those findings, a study of the cognitive effects of anesthetics in mice found that, after induction, the mice showed reduced alternation in the exploration of the arms in the Y maze and that miR-31 and miR-190b were significantly decreased in the medial prefrontal cortex and hippocampus [104].

Toyama et al. [103] studied the effects of vascular cognitive impairment in a model of bilateral common artery stenosis in mice and found that treatment with the microRNA inhibitor anti-miR-501-3p attenuated the spatial working memory deficits shown by the mice in the Y maze test [103]. Later, the same group studied community-dwelling Japanese older adults, some of whom presented comorbid diabetes mellitus or atherosclerosis, [78] and on the basis of the results hypothesized that vascular disruption contributes significantly to the burden of dementia. The authors evaluated the correlation between the serum expression of miR-501 measured by qPCR and the EF domain of the Montreal Cognitive Assessment and found that, regardless of the presence of a comorbid vascular condition, individuals with higher levels of miR-501 presented lower scores not only in EFs, but also in other cognitive domains, such as memory, attention, and visuospatial abilities.

Finally, for miR-9, Anderson et al. [97] found that after six months of an exercise intervention in older adults from the community, the levels of exosomal microRNA-9 were increased and associated with better performance in the Stroop A/C test [97]. Similarly, Gai et al. [112] showed that overexpression of miR-9-5p improved working memory in the hypoxia ischemia mouse model [112]. However, Malmevik et al. [108] inhibited the expression of three microRNAs in the hippocampi of mice including miR-9-5p by using a sponge design [108] and found no effects on the working memory performance of the mice in the Morris water maze test.

### Bioinformatic assessment of candidate microRNAs

**Fig. 3:**
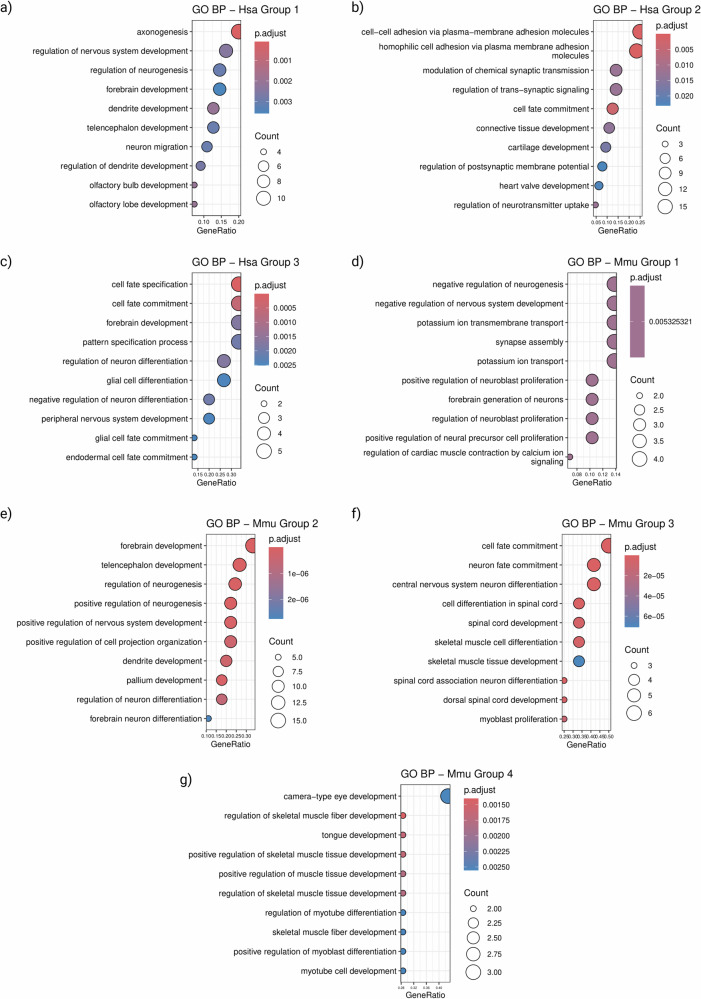
Analysis of microRNA dysregulation pathways associated with changes in executive functions. Pathways involved in the association of microRNA dysregulation and changes in executive functions (EFs) in the following groups: (**a**) microRNAs upregulated and EFs increased in humans; (**b**) microRNAs upregulated and EFs decreased in humans; (**c**) microRNA downregulated and EFs increased in humans; (**d**) microRNAs upregulated and EFs increased in rodents; (**e**) microRNAs upregulated and EFs decreased in rodents; (**f**) microRNA downregulated and EFs increased in rodents; (**g**) microRNA downregulated and EFs decreased in rodents.

The network analysis showed few overlaps between the identified gene hubs, with nodes ranging from 120 (Mouse group 1) to 943 (Human group 1). Common gene hubs were genes that regulate transcription or the cell cycle, such as MYC and EP300 [123, 124]. A summary is presented in eTable 10. In the pathway analysis, upregulated microRNAs were related to processes involving neurogenesis, neurodevelopment, and synaptic plasticity/signaling, whereas downregulated microRNAs were associated with cell/neuron fate commitment, spinal cord development/neuron differentiation, and peripheral nervous/motor processes. The 10 most significant pathways found in each group are presented in Fig. 3. An extended version is presented in the Supplement (eTable 11).

## Discussion

### Summary of the results

In this comprehensive review of the association between microRNA dysregulation and EFs in humans and animals, we found that alterations at the level of a microRNA or the related gene, loss of the microRNA biosynthesis machinery, and altered peripheral or tissue expression of microRNAs can either increase or decrease EFs. SNPs at the MIR137 gene were associated with reduced EFs. On the other hand, methylation in the promoter regions of the MIR885 gene was associated with successful cognitive aging. Loss of expression of the enzymes DGCR8 (22q11.2 DS) and DICER was associated with decreased working memory (a finding supported by electrophysiological findings). The consistently dysregulated microRNAs reported in two or more samples were as follows: in humans, miR-148a-3p; in rodents, miR-155, miR-30e, and miR-384-5p; and in both sample types, miR-132, miR-146a-5p, miR-148a-3p, miR-181a-5p, miR-190b, miR-31, miR-501-3p, and miR-9-5p. Gene ontology enrichment analysis showed that the targets of the dysregulated microRNAs were related to neurogenesis, neurodevelopment, and neuron fate commitment. Most of the human studies included patients with MCI or psychiatric conditions, and all of the studiesusing animal models evaluated only working memory. Our review thus identifies multiple potential targets on different levels that can be examined in future research, in particular in interventional studies aiming to improve higher-level cognition in humans.

### Gene changes in EF performance

The rs113948889 SNP at the MIR137 gene was associated with reduced EFs. Gene mutations in microRNA areas had been reported. For example, in the mice genome 1700 SNPs were present in 702 pre-microRNAs and 609 SNPs in mature microRNAs and 841 SNPs could destabilize the secondary structure of the corresponding microRNA [125]. Also, a study in schizophrenia patients reported that cognitive impairment, including executive dysfunction, was longitudinally persistent and correlated with cortical structural changes, mostly in the frontal and temporal lobes [126]. In line with those results, a mega-analysis of GWASs on schizophrenia found that the candidate gene MIR137 was related to the pathology of the disease [127], and various authors replicated its association with the diagnosis of schizophrenia in independent studies [128]. VNTRs in this gene were found to be associated with morphological changes in the brain, such as a thicker left inferior temporal gyrus and deeper right postcentral sulcus, and with a cognitive deficit subtype of schizophrenia [129]. Other gene mutations may also be of interest and require future research.

### Nuclear microRNA processing

The 22q11.2 deletion syndrome and the associated dysregulation by microRNAs may explain the associated high risk for schizophrenia, a psychiatric disorder strongly linked with deficient EFs [130]. The syndrome is characterized by a genetic mutation in the area closely related to the expression of the DGCR enzyme, which is needed for the biosynthesis of microRNAs. Different microRNAs have been reported as dysregulated in 22q11.2 DS, but a clear signature is still lacking [131]. Interestingly, patients with this syndrome have a high prevalence of various psychiatric disorders: As many as 70% of them have anxiety, depression, or attention-deficit/hyperactivity disorder and up to 25% have schizophrenia [34]. Impairment of EFs and other cognitive disabilities are also part of the phenotype [132]. These findings imply the potential role of microRNAs in the normal development of the brain and in maintaining optimal higher brain functioning.

### DICER

In an inflammatory model resembling Parkinson disease in microglia and mice, one study showed that microglial DICER degradation is associated with increased inflammation and dopaminergic neuronal loss [133]. To this end, a physical exercise intervention in 3xTg mice (an Alzheimer disease model) showed that after 20 weeks, in the active group the levels of DICER and miR-29 were higher and the levels of the latter correlated with lower levels of one of its targets, Beta-secretase 1. This result might suggest that β−amyloid accumulation is related to lower levels of DICER [134]. Another experiment deleted the Dicer1 gene in the adult mouse forebrain and found downregulation of various microRNAs. After 12 weeks, the authors observed an increase in memory performance as a prodrome of neurodegeneration, suggesting the importance of microRNAs for cognitive processes in general [135].

### Differential expression

The microRNAs that were reported as consistently dysregulated in two or more samples and had an inverse correlation with EFs were miR-155 and miR-30e in rodents and miR-132, miR-146a-5p, miR-148a-3p, miR-181a-5p, and miR-501-3p in both samples. MiR-384-5p presented a direct correlation with EFs in rodents, and miR-190b, miR-31, and miR-9-5p did so in both sample types.

A systematic review that included a meta-analysis of 147 different datasets on differential expression of microRNAs in Alzheimer disease patients found similar results regarding these microRNAs. [136] In line with our findings, the authors reported that across the studies, hsa-miR-501-3p was consistently upregulated in the brains of these patients. Additionally, miR-146a-5p was reported as upregulated in brain and downregulated in blood, which partially agrees with our results and highlights the tissue specificity of microRNA expression. Moreover, these findings suggest that because microRNA signatures are related to MCI, they could also serve as a predictor of an early stage of Alzheimer disease, justifying further research.

Another systematic review that evaluated microRNAs dysregulated in association with neurodegenerative disorders included 641 studies and identified similar microRNAs among the most frequently occurring candidates [33]. Like the present study, the review found that miR-146a-5p and miR-155-5p were upregulated in patients with neurogenerative disorders. Impairment of EFs is a common feature of neurodegenerative disorders [137], and the authors proposed that dysregulation of these microRNAs could affect shared pathways related to cognition, including an increase in proinflammatory markers, increased amyloid beta genesis, and altered neurogenesis and neural cell differentiation [33].

In addition to studying microRNAs, evaluating other classes of small non-coding RNAs could improve our understanding of the epigenetic mechanisms behind EFs. One such class is transfer RNA-derived small fragments (tRFs), which are produced through the cleavage of precursor or mature transfer RNAs, often in response to cellular stress. [138] This emerging class of small non-coding RNAs owes its diversity to the fact that tRFs can arise from precursor or mature transfer RNAs. [139] These fragments are not only diverse in origin, but also abundant in various biological fluids: They make up a large proportion of small RNAs in urine (84%), amniotic fluid (75%), and cerebrospinal fluid ( ~ 47%). [140] The roles of tRFs are similar to those of microRNAs in that they are capable of binding Argonaute proteins to silence the expression of mRNA. [141] Also noteworthy is that given their higher levels in neurons and their higher occurrence relative to microRNAs, tRFs likely play a broader role in regulating gene networks associated with EF-related processes. Further support for this hypothesis is provided by the finding that their dysregulation is linked to neurological disorders such as epilepsy, amyotrophic lateral sclerosis, and cognitive decline in Alzheimer disease. [142–144] The major role of EFs in healthy cognition and their sensitivity to brain disorders supports the hope that further research on tRFs could uncover new mechanisms and gene targets related to this phenotypic trait.

### Bioinformatics analysis of microRNA targets

According to our exploratory bioinformatic analysis, the most relevant pathways through which microRNA regulation influences EFs include neurogenesis, nervous system development, and synapse regulation and signaling.

Adult neurogenesis was first observed in rodent models and was later confirmed in the adult human hippocampus (dentate gyrus) to be responsible for lifelong synaptic plasticity. [145–147] Alterations in neurogenesis that occur during aging or in neuropsychiatric conditions can impact executive functioning, hence producing impaired cognitive flexibility and working memory [148]. This process is susceptible to environmental modifications such as enrichment [149], but epigenetic regulations also play an important role. For instance, increased levels of miR-132, a candidate that was identified also in this review, improved memory deficits in a mouse model of Alzheimer disease by rescuing hippocampal neurogenesis. [150] On the other hand, hippocampal upregulation of miR-146-5p was associated with higher cognitive impairment, whereas its downregulation increased hippocampal neurogenesis and rescued the phenotype. [151, 152] In the case of the miR-17-92 cluster, inhibition of its expression in a transgenic mouse model led to reduced hippocampal neurogenesis and a phenotype of cognitive impairment [153], which was later observed to be associated with symptoms of anxiety and depression [154].

Regulation by microRNAs also takes place during neurodevelopment, including forebrain development, neuronal differentiation and maturation, and synaptogenesis [155]. One example is miR-124, which regulates neuronal development by targeting the Notch pathway. [156] Further study of the involvement of these and other mechanisms in the regulation of EFs by microRNA candidates is granted.

Synaptic plasticity in the prefrontal cortex (an area relevant to EFs) is mediated by dopamine signaling and explains to some extent differences in the performance of rodents in cognitive tasks [157]. MicroRNAs regulate synaptic plasticity by silencing relevant plasticity mediators and dendritic proteins needed for signal transmission [158]. For example, inhibition of microRNA 135a-5p expression in mice produces synaptic dysfunction and memory impairment [157]. In the case of major depressive disorder, several microRNAs interfere with synaptic signaling and protein-synthesis dependent synaptic plasticity [159]. These mechanisms could potentially explain cognitive deficits in major depressive disorder and other psychiatric disorders.

## Conclusions and future perspectives

Alterations of microRNA biosynthesis at the gene level, loss of the microRNA biosynthesis machinery, and altered peripheral or tissue-specific expression of microRNAs affect EFs. These changes mostly reduce EFs, but some improvement was also observed. A total of eight candidates were found consistently across sample types (i.e., in both human and rodent studies). The targets of the significantly dysregulated microRNAs were related to common pathways such as neurogenesis, neurodevelopment, and synaptic plasticity. Although it was not the focus of this review, isoforms of miRNAs (isomiRs) which are variants of the canonical from of different sequences or length, improve the specificity of target regulation and have been explored to improve the precision of miRNA-based biomarkers, [160] showing promising results in animal models of epilepsy. [161]

Most of the human studies were in patients with MCI or psychiatric conditions, and all of the animal studies evaluated only working memory. These microRNA signatures require further confirmation as potential biomarkers in human studies with larger sample sizes and in controlled and blinded interventions. If confirmed as such, these signatures could not only be easily implemented in clinical practice to classify at-risk patients and provide additional cognitive training, but they could also be considered as therapeutic targets to enhance EFs or prevent their decline. Therefore, research on this topic is necessary to improve the quality of life of patients with psychiatric, neurologic, and inflammatory disorders and of healthy aging individuals. Additionally, longitudinal studies could provide a broader perspective of the relationship between microRNA expression and EFs, both of which are dynamic constructs, to identify predictive signatures that correlate with the various trajectories of EFs.

## Limitations

The high heterogeneity resulting from the various methodological approaches used in the studies (i.e., selection of different diagnoses, use of different microRNA processing methods and bioinformatic pipelines, and differences in statistical analysis methods) prevented us from evaluating the effects related to the commonly dysregulated microRNAs by a more detailed analysis, such as a pooled meta-analysis.

Most of the included studies had a moderate to high risk of bias. Human cohorts were deficient in the representativeness of the sample chosen, blinding of the personnel involved in data processing and analysis, and the longitudinal assessment of the outcomes.

This review identified mostly cross-sectional human studies assessing miRNA expression. Only two studies used a longitudinal approach, but no repeated measures of microRNA expression in humans related to executive functions were found. Cross-sectional designs can establish associations between miRNA expression and executive function phenotypes but cannot infer causality due to temporal ambiguity, reverse causation, and confounding. Longitudinal studies tracking miRNA dynamics over time, paired with cognitive assessments, are needed to elucidate directional relationships and rule out reverse causality (e.g., cognitive decline influencing miRNA expression).

In most of the case-control studies, sample sizes were reduced, non-responders were not clearly described, assessors were not blinded, and the comparability of the sample with other studies was decreased because of the type of statistical analyses performed. Animal studies lacked randomization of housing, blinding of outcome assessment, and a registered or publicly available protocol, all of which could limit the reliability and validity of the results.

Our bioinformatics pipeline presented limitations which are common to miRNA research. The in-house tool aggregated experimentally validated targets from NPInter (ncRNA-protein/RNA), TarBase (miRNA-mRNA), TransmiR (transcription factor-miRNA), RegNetwork (regulatory networks), Rise (regulatory interaction search), and STRING (protein interactions). While validated targets provide higher confidence, they present limited coverage with an overrepresentation of studies in the field of cancer. [162] These limitations propagate to the pathway enrichment analysis, where Gene Ontology over-representation can reflect biological processes biased towards that field. [163] We therefore interpret networks and pathways as hypothesis-generating at the module level, not as confirmed direct 3′UTR/promoter interactions. Definitive validation requires future testing with functional assays (e.g., 3′UTR luciferase reporters with mutagenesis, CLIP). The above-mentioned limitations should be taken into consideration when interpreting the results of this review.

## Supplementary information

Supplement
