# Supplementary material for: The role of microRNAs in executive functions: a comprehensive review and bioinformatics analysis of human and animal studies
Source: Mol Psychiatry. 2026 May 1;31(9):5348–72. doi: 10.1038/s41380-026-03623-2 (PMC13441878; doi:10.1038/s41380-026-03623-2)
Supplement: Supplementary file 1 [file 41380_2026_3623_MOESM1_ESM.docx]

This is the supplementary material from

**The role of microRNAs in executive functions: A comprehensive review and bioinformatics analysis of human and animal studies**

Alba Navarro-Flores, Dennis M. Krüger, Lalit Kaurani, Andre Fischer, Thomas G Schulze, &

Urs Heilbronner

**Content**

[**eTable1.**	**PRISMA 2020 Checklist**	2](#_Toc201318734)

[**eTable2.**	**Search Strategy**	4](#_Toc201318735)

[**eTable3.**	**Risk of bias criteria - SYRCLE**	5](#_Toc201318736)

[**eTable4.**	**Risk of bias criteria – New Castle Ottawa for Cohorts**	7](#_Toc201318737)

[**eTable5.**	**Risk of bias criteria – New Castle Ottawa for Case-Controls**	8](#_Toc201318738)

[**eTable6.**	**Risk of bias criteria - RCT**	9](#_Toc201318739)

[**eTable7.**	**Excluded studies with reasons**	11](#_Toc201318740)

[**eTable8.**	**Risk of bias assessment – human cohorts**	14](#_Toc201318741)

[**eTable9.**	**Risk of bias assessment – human case-control studies**	14](#_Toc201318742)

[**eTable10.**	**Summary of the Network Analysis of the microRNAs’ gene targets**	15](#_Toc201318743)

[**eFigure2.**	**Risk of bias traffic light plot – Animal studies “SYRCLE tool”**	16](#_Toc201318744)

[**eFigure3.**	**Risk of bias summary plot – Animal studies “SYRCLE tool”**	17](#_Toc201318745)

[**eFigure4.**	**Risk of bias traffic light plot – Human Studies “RoB2 tool”**	17](#_Toc201318746)

[**eTable10.**	**Biological processes found after gene ontology enrichment analysis**	18](#_Toc201318747)

[**eReferences**	25](#_Toc201318748)

eTable1. **PRISMA 2020 Checklist**

| **Section and Topic** | **Item #** | **Checklist item** | **Page of item´s report** |  |
| --- | --- | --- | --- | --- |
| **TITLE** | | | | |
| Title | 1 | Identify the report as a systematic review. | 01 |  |
| **ABSTRACT** | | | | |
| Abstract | 2 | See the PRISMA 2020 for Abstracts checklist. | 02 |  |
| **INTRODUCTION** | | | | |
| Rationale | 3 | Describe the rationale for the review in the context of existing knowledge. | 03-04 |  |
| Objectives | 4 | Provide an explicit statement of the objective(s) or question(s) the review addresses. | 04-05 |  |
| **METHODS** |  |  |  |  |
| Eligibility criteria | 5 | Specify the inclusion and exclusion criteria for the review and how studies were grouped for the syntheses. | 05 |  |
| Information sources | 6 | Specify all databases, registers, websites, organisations, reference lists and other sources searched or consulted to identify studies. Specify the date when each source was last searched or consulted. | 05 |  |
| Search strategy | 7 | Present the full search strategies for all databases, registers and websites, including any filters and limits used. | Supp |  |
| Selection process | 8 | Specify the methods used to decide whether a study met the inclusion criteria of the review, including how many reviewers screened each record and each report retrieved, whether they worked independently, and if applicable, details of automation tools used in the process. | 05-06 |  |
| Data collection process | 9 | Specify the methods used to collect data from reports, including how many reviewers collected data from each report, whether they worked independently, any processes for obtaining or confirming data from study investigators, and if applicable, details of automation tools used in the process. | 05 |  |
| Data items | 10a | List and define all outcomes for which data were sought. Specify whether all results that were compatible with each outcome domain in each study were sought (e.g. for all measures, time points, analyses), and if not, the methods used to decide which results to collect. | 05 |  |
|  | 10b | List and define all other variables for which data were sought (e.g. participant and intervention characteristics, funding sources). Describe any assumptions made about any missing or unclear information. | 05-06 |  |
| Study risk of bias assessment | 11 | Specify the methods used to assess risk of bias in the included studies, including details of the tool(s) used, how many reviewers assessed each study and whether they worked independently, and if applicable, details of automation tools used in the process. | 06 |  |
| Effect measures | 12 | Specify for each outcome the effect measure(s) (e.g. risk ratio, mean difference) used in the synthesis or presentation of results. | NA |  |
| Synthesis methods | 13a | Describe the processes used to decide which studies were eligible for each synthesis (e.g. tabulating the study intervention characteristics and comparing against the planned groups for each synthesis (item #5)). | 06 |  |
|  | 13b | Describe any methods required to prepare the data for presentation or synthesis, such as handling of missing summary statistics, or data conversions. | 06 |  |
|  | 13c | Describe any methods used to tabulate or visually display results of individual studies and syntheses. | 06 |  |
|  | 13d | Describe any methods used to synthesize results and provide a rationale for the choice(s). If meta-analysis was performed, describe the model(s), method(s) to identify the presence and extent of statistical heterogeneity, and software package(s) used. | 06 |  |
|  | 13e | Describe any methods used to explore possible causes of heterogeneity among study results (e.g. subgroup analysis, meta-regression). | NA |  |
|  | 13f | Describe any sensitivity analyses conducted to assess robustness of the synthesized results. | NA |  |
| Reporting bias assessment | 14 | Describe any methods used to assess risk of bias due to missing results in a synthesis (arising from reporting biases). | 06 |  |
| Certainty assessment | 15 | Describe any methods used to assess certainty (or confidence) in the body of evidence for an outcome. | NA |  |
| **RESULTS** |  |  |  |  |
| Study selection | 16a | Describe the results of the search and selection process, from the number of records identified in the search to the number of studies included in the review, ideally using a flow diagram. | 07 |  |
|  | 16b | Cite studies that might appear to meet the inclusion criteria, but which were excluded, and explain why they were excluded. | Supp |  |
| Study characteristics | 17 | Cite each included study and present its characteristics. | 07/Tab1-2 |  |
| Risk of bias in studies | 18 | Present assessments of risk of bias for each included study. | Supp |  |
| Results of individual studies | 19 | For all outcomes, present, for each study: (a) summary statistics for each group (where appropriate) and (b) an effect estimate and its precision (e.g. confidence/credible interval), ideally using structured tables or plots. | NA |  |
| Results of syntheses | 20a | For each synthesis, briefly summarise the characteristics and risk of bias among contributing studies. | NA |  |
|  | 20b | Present results of all statistical syntheses conducted. If meta-analysis was done, present for each the summary estimate and its precision (e.g. confidence/credible interval) and measures of statistical heterogeneity. If comparing groups, describe the direction of the effect. | NA |  |
|  | 20c | Present results of all investigations of possible causes of heterogeneity among study results. | NA |  |
|  | 20d | Present results of all sensitivity analyses conducted to assess the robustness of the synthesized results. | NA |  |
| Reporting biases | 21 | Present assessments of risk of bias due to missing results (arising from reporting biases) for each synthesis assessed. | 08/Supp |  |
| Certainty of evidence | 22 | Present assessments of certainty (or confidence) in the body of evidence for each outcome assessed. | NA |  |
| **DISCUSSION** | | | | |
| Discussion | 23a | Provide a general interpretation of the results in the context of other evidence. | 16-19 |  |
|  | 23b | Discuss any limitations of the evidence included in the review. | 20 |  |
|  | 23c | Discuss any limitations of the review processes used. | 20 |  |
|  | 23d | Discuss implications of the results for practice, policy, and future research. | 19-20 |  |
| **OTHER INFORMATION** | | | | |
| Registration and protocol | 24a | Provide registration information for the review, including register name and registration number, or state that the review was not registered. | NA |  |
|  | 24b | Indicate where the review protocol can be accessed, or state that a protocol was not prepared. | NA |  |
|  | 24c | Describe and explain any amendments to information provided at registration or in the protocol. | NA |  |
| Support | 25 | Describe sources of financial or non-financial support for the review, and the role of the funders or sponsors in the review. | 21 |  |
| Competing interests | 26 | Declare any competing interests of review authors. | 21 |  |
| Availability of data, code and other materials | 27 | Report which of the following are publicly available and where they can be found: template data collection forms; data extracted from included studies; data used for all analyses; analytic code; any other materials used in the review. | Supp |  |

Adapted from: Page MJ, McKenzie JE, Bossuyt PM, Boutron I, Hoffmann TC, Mulrow CD, et al. The PRISMA 2020 statement: an updated guideline for reporting systematic reviews. BMJ 2021;372:n71. doi: 10.1136/bmj.n71

For more information, visit: http://www.prisma-statement.org/

eTable2. **Search Strategy**

Date 18 October 2023

| Database | Search strategy | N° |
| --- | --- | --- |
| Pubmed/Medline | (microrna[tiab] or "miR-"[tiab] or "mirna"[tiab]) and ("executive functions"[tiab] or "executive function"[tiab] or "executive functioning"[tiab] or "working memory"[tiab] or "trail making"[tiab] or "digit spam"[tiab] or "set shifting"[tiab] or "cognitive decline"[tiab] or neuropsycholog*[tiab]) | 218 |
| Scopus | ( TITLE-ABS-KEY ( "microrna" ) OR TITLE-ABS-KEY ( "mirna" [tiab] ) ) AND ( TITLE-ABS-KEY ( "executive functions" ) ] OR TITLE-ABS-KEY ( "working memory" [tiab] ) OR TITLE-ABS-KEY ( "trail making" [tiab] ) OR TITLE-ABS-KEY ( "digit spam" ) OR TITLE-ABS-KEY ( "set shifting" ) OR TITLE-ABS-KEY ( "cognitive decline" ) ) | 245 |
| Web of Science | (TI=(microrna) OR AB=(microrna) OR TI=(micro rna) OR AB=(micro rna) OR TI=(mirna) OR AB=(mirna)) AND (TI=(“executive function”) OR AB=(“executive function”) OR TI=(“executive functions”) OR AB=(“executive functions”) OR TI=(“working memory”) OR AB=(“working memory”) OR TI=(“trail making”) OR AB=(“trail making”) OR TI=(“digit spam”) OR AB=(“digit spam”) OR TI=(“set shifting”) OR AB=(“set shifting”) OR TI=(“cognitive decline”) OR AB=(“cognitive decline”)) | 127 |
| Total records |  | 590 |
| Duplicates |  | 180 |
| Total for screening |  | 410 |

eTable3. **Risk of bias criteria - SYRCLE**

| **Item** | **Signaling Questions** | **Decisions** |
| --- | --- | --- |
| Selection bias |  |  |
| Item 1: Sequence generation | Did the investigators describe a random component in the sequence generation process? | ***Yes:*** Explicitly saying: Random number table, random number computer generator or other appropriate randomization methods.  ***Unclear:*** Randomization process mentioned, but not further information was given.  ***No:*** Explicitly stating that no randomization was done, or done with not appropriate methods as by judgment or by investigator´s preference, based on results of laboratory tests, availability of the intervention, by odd or date of birth, animal number or cage number. Or not reported. |
| Item 2: Baseline Characteristics | Was the distribution of relevant baseline characteristics balanced for the intervention and control groups? | ***Yes:*** They mentioned sex, age and weight of the animals were within a narrow range in both groups. Or mentioned they come from the same provider.  ***Unclear:*** Weight or other relevant variables not reported, brought from different laboratories.  ***No:*** Baseline characteristics were differently distributed. |
|  | If relevant, did the investigators adequately adjust for unequal distribution of some relevant baseline characteristics in the analysis? | ***NA:*** Adjustment not relevant  Yes: Adjust for variables that are unbalanced and different.  Unclear: Not adjustment but not clear if needed.  No: No adjustment but it was needed. |
|  | **Was the timing of disease induction adequate? (separate item?)** | **Yes:** The disease was induced before randomization of the intervention. The disease was induced after randomization of the intervention, but the timing of disease induction was at random, and the individual inducing the disease was adequately blinded from knowing which intervention each animal received. Evaluated case by case. |
| Item 3: Allocation concealment | Could the investigator allocating the animals to intervention or control group not foresee assignment due to one of the following or equivalent methods? | ***Yes:*** Allocation concealment was performed sufficiently (e.g., Third-party coding of experimental and control group allocation; Central randomization by a third party; Sequentially numbered opaque, sealed envelopes.)  ***Unclear:*** Insufficient allocation concealment, or with unclear methodology (Open randomization schedule, Envelopes without appropriate safeguard, Alternation or rotation, Allocation based on date of birth, or on animal number, Any other explicitly unconcealed procedure). Not report of allocation concealment in the manuscript.  ***No:*** Explicitly mentioned that allocation concealment was not done. |
| **Performance bias:** | | |
| Item 4: Random Housing | *Did the authors randomly place the cages or animals within the animal room/facility? Animals were selected at random during outcome assessment (use signaling questions of entry 6) Yes: at least some description of housing. | ***Yes:*** Explicitly stating that randomization in the animal facility was conducted.  ***Unclear*** (insufficient information): The animals from the various experimental groups live together in one cage/pasture (e.g., housing conditions are identical).  ***No:*** Explicitly stating that no randomization was performed. Experimental groups were studied on various locations (e.g., group A in lab A or on shelf A; Group B in Lab B or on shelf B). No report of animal facilities. |
| Item 5: Blinding while caring | *Was blinding of caregivers and investigators ensured, and was it unlikely that their blinding could have been broken? | ***Yes:*** Blinding was performed in the caregivers and sufficiently (ID cards of individual animals, or cage/animal labels are coded and identical in appearance, etc.)  ***Unclear:*** Blinding was performed insufficiently.  ***No:*** Binding not performed or not mentioned. |
| **Detection bias** | | |
| Item 6: Random outcome assessment | Did the investigators randomly pick an animal during outcome assessment, or did they use a random component in the sequence generation for outcome assessment? | ***Yes:*** Outcome was assessed randomly and appropriately (referring to a random number table, computer random number generator)  ***Unclear:*** Outcome was assessed randomly but not appropriately or no info about the methods was mentioned.  ***No:*** Explicitly mentioning that it was not randomly assessed or no information about it reported. |
| Item 7: Blinding while assessing | Was blinding of the outcome assessor ensured, and was it unlikely that blinding could have been broken? | ***Yes:*** Investigator mentioned as blinded to groups.  ***Unclear:*** The investigator was not blinded but the outcomes´ assessment was aided with technology as videotaping.  ***No:*** Explicitly mentioning that it was not randomly assessed or no information about it reported. |
| **Attrition bias** | | |
| Item 8 | Were all animals included in the analysis? | Yes: same number at the beginning and at the end of the experiment. (no report of outliers) /  Unclear: The number of the baseline or post-intervention animals were not reported.  No: Not all animals were included in the analysis. |
|  | Were the reasons for missing outcome data unlikely to be related to true outcome? (e.g., technical failure) | Yes  No  Unclear |
|  | Are missing outcome data balanced in numbers across intervention groups, with similar reasons for missing data across groups? | Yes  No  Unclear |
|  | Are missing outcome data imputed using appropriate methods? | Yes  No  Unclear  NA |
|  |  | The decision was made with the average of this responses. |
| **Reporting bias** | | |
| Item 9: Selective outcome reporting | Was the study protocol available and were all of the study’s pre-specified primary and secondary outcomes reported in the current manuscript? | Yes: Protocol available.  Unclear: Protocol mentioned but not available.  No: Protocol not mentioned. |
| **Other** | | |
| Item 10: Other sources of bias | Is it unlikely to have other biases?  We considered funding source as a potential additional bias. | Yes: Funding reported and only from academic sources.  Unclear: Funding from Industry and potential conflicts of interest.  No: Funding or conflicts of interest not reported. |

**Legend.** NA was written when an item did not apply in the context of a particular study design e.g. one-arm experiments. These criteria were adopted from the original manuscript ^1^, and suggestion were taken from a previously published review were used ^2^.

eTable4. **Risk of bias criteria – New Castle Ottawa for Cohorts**

| **Item** | **Criteria** |
| --- | --- |
| **Selection** |  |
| 1) Representativeness of the exposed cohort | a) Truly representative (one star): community sample and random or census sampling.  b) Somewhat representative (one star): institutional with any sampling, or a convenience sample. And the sample size more than minimum (n=385*), unless sample size calculation stated.  c) Selected group: other specific population, or sample size lower than 385.  d) No description |
| 2) Selection of the non-exposed cohort (the patients without the genotype of interest) | a) Drawn from the same community as the exposed cohort (one star)  b) Drawn from a different source  c) No description of the derivation of the non-exposed cohort |
| 3) Ascertainment of exposure (The genotype/ differential expression in microRNA studies) | a) From a laboratory analysis (two stars): qPCR, NGS, etc.  b) Derived from in-silico predictions (one star)  c) Written self-report  d) No description  e) Other |
| 4) Demonstration that outcome of interest was not present at start of study | Because the time for the effects of genetic alterations related to cognition cannot be predicted, this item did not apply and was not used. |
| **Comparability** |  |
| 5) Comparability of cohorts on the basis of the design or analysis controlled for confounders | a) The study controls for age, sex and education years (one star) - at least two of them  b) Study controls for other factors (one star)  c) Cohorts are not comparable on the basis of the design or analysis not controlled for confounders – or not reported |
| **Outcome** |  |
| 6) Assessment of outcome | a) Independent blind assessment (one star)  b) Record linkage (one star)  c) Self report or unblinded  d) No description  e) Other |
| 7) Was follow-up long enough for outcomes to occur - only when needed | a) Yes (one star): "more than 3 months"  b) No  c) Not applicable |
| 8) Adequacy of follow-up of cohorts (also response rate in cross-sectionals) | a) Complete follow up- all subject accounted for (one star)  b) Subjects lost less than or equal to 20% or description of those lost suggested no different from those followed. (one star)  c) Follow up rate less than 80% and no description of those lost, or no comparable  d) No statement |

**Legend.** * We calculated the minimum sample size using an online open access tool (<https://www.calculator.net/sample-size-calculator.html>), with the following parameters: Confidence level 95%, margin of error 5%, population proportion of 50% (we used a proportion of 50% since the prevalence of executive function decline in various groups has not been determined before), and leaving blank the population size.

eTable5. **Risk of bias criteria – New Castle Ottawa for Case-Controls**

| **Item** | **Criteria** |
| --- | --- |
| **Selection** |  |
| 1) Representativeness of the sample | a) Truly representative of the average in the target population (one star): all subjects or random sampling.  b) Somewhat representative of the average in the target population (one star): nonrandom sampling.  c) Selected group of users.  d) No description of the sampling strategy. |
| 2) Sample size | a) Justified and satisfactory (one star): a threshold of >= 385 * or if less offering a justification (sample size calculation).  b) Not justified. - the other options |
| 3) Non-respondents | a) Comparability between respondents and non-respondents’ characteristics is established, and the response rate is satisfactory (>80%): one star  b) The response rate is unsatisfactory, or the comparability between respondents and non-respondents is unsatisfactory. |
| 4) Validated measurement tool | a) From a laboratory analysis (two stars): qPCR, NGS, etc.  b) Derived from in-silico predictions (one star)  c) Written self-report  d) No description  e) Other |
| **Comparability** |  |
| 5) Comparability of the samples | a) The study controls for the most important factor (one star): use of regression models (or correlations) to control for sex, age, education year (at least 2 of those variables).  b) The study control for any additional factor (one star): Use of regression models to control for other variables i.e., education years.  c) Neither |
| **Outcome** |  |
| 6) Assessment of outcome | a) Independent blind assessment (two stars): Sequencing, qPCR, or automatized genotyping, ideally with blinded researchers.  b) Record linkage (two star): same but from a secondary source i.e., a different study.  c) Unblinded (one star): sequencing, qPCR, other biological confirmation methods but unblinded.  d) No description / other |
| 7) Statistical test | a) The statistical test used to analyze the data is clearly described and appropriate, and the measurement of the association is presented, including confidence intervals and the probability level (p value): one star  b) The statistical test is not appropriate, not described or incomplete. |

**Legend.** * We calculated the minimum sample size using an online open access tool (<https://www.calculator.net/sample-size-calculator.html>), with the following parameters: Confidence level 95%, margin of error 5%, population proportion of 50% (we used a proportion of 50% since the prevalence of executive function decline in various groups has not been determined before), and leaving blank the population size.

eTable6. **Risk of bias criteria - RCT**

The RoB 2.0 tool ^3^ evaluates risk of bias of RCTs in five domains:

| **Item** | **Criteria** |
| --- | --- |
| (a) randomization process | 1. Random sequence allocation  2. Appropriate sequence concealment  3. Are there baseline differences between groups which might suggests issues with the randomization process? |
| (b) intervention process | 1. Awareness of the assigned intervention from participants  2. Awareness of the assigned intervention from caregivers and people delivering the intervention  3. Presence of deviations from the intended intervention  4. The deviations were likely to affect the outcome  5. The deviations were balanced among groups.  6. There was an appropriate analysis to estimate the effect of the assignment in the intervention.  7. The failure to analysis participants in the corresponding group might impact the results. |
| (c) missing outcome data | 1. The data for the outcome of interest was available for all the participants (or almost all)  2. The results were not biased due to the missing data  3. The reason of missing data depends on the true value of the data.  4. It is very likely that 3 is true. |
| (d) measurement of the outcome | 1. Appropriate methods for measuring the outcome  2. It is possible that measurements of the outcome differed between intervention groups.  3. Outcome assessors were aware of the intervention.  4. It is possible that outcome assessment was influences by the knowledge of the received intervention.  5. It is very likely that 4 is true |
| (e) selection of the reported result | 1. The data from this result was analyzed according to a pre-specified plan which was completed before the unblinding of the data.  It is likely that the numerical result was selected after  2. Multiple eligible outcome measurements within the domain.  3. Multiple eligible analysis of the data. |

**Legend.** Categorization into low, high, and unclear risk of bias was conducted according to the Cochrane handbook.^4^

eFigure1. **Flowchart of the selection process**

eTable7. **Excluded studies with reasons**

| **Study ID** | **Title** | **Reason** |
| --- | --- | --- |
| Abuelezz et al (2022) | Sera miR-34a, miR-29b and miR-181c as potential novel diagnostic biomarker panel for Alzheimers in the Egyptian population | Executive functions not an outcome |
| Abyadeh et al (2023) | Common Molecular Signatures Between Coronavirus Infection and Alzheimer's Disease Reveal Targets for Drug Development | Executive functions not an outcome |
| Agostini et al (2019) | Serum miRNAs Expression and SNAP-25 Genotype in Alzheimer's Disease | Executive functions not an outcome |
| Al-Rawaf (2021) | Molecular Changes in Circulating microRNAs' Expression and Oxidative Stress in Adults with Mild Cognitive Impairment: A Biochemical and Molecular Study | Executive functions not an outcome |
| Che et al (2014) | Expression of amyloid-associated miRNAs in both the forebrain cortex and hippocampus of middle-aged rat | Executive functions not an outcome |
| Chen et al (2019) | MicroRNA-146a protects against cognitive decline induced by surgical trauma by suppressing hippocampal neuroinflammation in mice | Executive functions not an outcome |
| Cheng et al (2015) | Prognostic serum miRNA biomarkers associated with Alzheimer's disease shows concordance with neuropsychological and neuroimaging assessment | Executive functions not an outcome |
| Chow et al (2018) | Identification of AKT1 3′UTR variants in two Indian schizophrenia patients with poor executive functioning | Letter to the editor |
| Comfort et al (2022) | Extracellular microRNA and cognitive function in a prospective cohort of older men: The Veterans Affairs Normative Aging Study | Executive functions not an outcome |
| Cong et al (2014) | Expression of early growth response 1 affects miR-106a/signal transducer and activator of transcription 3 regulating cognitive impairment in ovariectomized mice | Executive functions not explicitly evaluated |
| Cosin-Tomas (2018) | Temporal Integrative Analysis of mRNA and microRNAs Expression Profiles and Epigenetic Alterations in Female SAMP8, a Model of Age-Related Cognitive Decline | Executive functions not an outcome |
| Cosin-Tomas (2014) | Epigenetic alterations in hippocampus of SAMP8 senescent mice and modulation by voluntary physical exercise | Executive functions not an outcome |
| Cui et al (2018) | Exosomes derived from hypoxia-preconditioned mesenchymal stromal cells ameliorate cognitive decline by rescuing synaptic dysfunction and regulating inflammatory responses in APP/PS1 mice | Executive functions not an outcome |
| Dakterzada et al (2021) | Reduced Levels of miR-342-5p in Plasma Are Associated With Worse Cognitive Evolution in Patients With Mild Alzheimer's Disease | Executive functions not an outcome |
| De Felice et al (2020) | MicroRNA Expression Signature in Mild Cognitive Impairment Due to Alzheimer's Disease | Executive functions not an outcome |
| Deng et al (2023) | miR-146a-5p Modulates Adult Hippocampal Neurogenesis Deficits Through Klf4/p-Stat3 Signaling in APP/PS1 Mice | Executive functions not an outcome |
| Dong et al (2023) | MicroRNA-132 is associated with the cognition improvement following voluntary exercise in SAMP8 mice | Executive functions not an outcome |
| Dragovic et al (2022) | Inflammation-Related microRNAs-146a and -155 Are Upregulated in Mild Cognitive Impairment Subjects among Older Age Population in Montenegro | Executive functions not an outcome |
| Hosseininia et al (2023) | Memory impairment was ameliorated by corticolimbic microinjections of arachidonylcyclopropylamide (ACPA) and miRNA-regulated lentiviral particles in a streptozotocin-induced Alzheimer's rat model | Executive functions not an outcome |
| Huang et al (2020) | Sex difference in cognitive impairment in drug-free schizophrenia: Association with miR-195 levels | Executive functions not an outcome |
| Ishihara et al (2021) | Serum levels of miRNA-221 and-222 may be predictive biomarkers for cognitive decline | Abstract |
| Kenny et al. (2019) | Elevated Plasma microRNA-206 Levels Predict Cognitive Decline and Progression to Dementia from Mild Cognitive Impairment | Executive functions not an outcome |
| Kim et al. (2022) | Identification of miRNA expression associated with Alzheimer's disease and neurodegeneration in rat models with obstructive sleep apnea | Executive functions not an outcome |
| Kovalchuk et al. (2017) | Growth of malignant extracranial tumors alters microRNAome in the prefrontal cortex of TumorGraft mice | Executive functions not an outcome |
| Kubota et al. (2018) | Involvement of microRNA-134 in the working memory-enhancing effect of nicotinic acetylcholine receptor agonist | Book chapter |
| Li N. et al. (2023) | Remote ischemic conditioning alleviates chronic cerebral hypoperfusion-induced cognitive decline and synaptic dysfunction via the miR-218a-5p/SHANK2 pathway | Executive functions not an outcome |
| Li T. et al. (2023) | Cognitive–exercise dual-task intervention ameliorates cognitive decline in natural aging rats via inhibiting the promotion of LncRNA NEAT1/miR-124–3p on caveolin-1-PI3K/Akt/GSK3β Pathway | Executive functions not an outcome |
| Li Y. et al (2022) | Circ-Vps41 positively modulates Syp and its overexpression improves memory ability in aging mice | Executive functions not an outcome |
| Liu C. et al (2022) | Identification of potential key circular RNAs related to cognitive impairment after chronic constriction injury of the sciatic nerve | No microRNA analysis |
| Liu Y. et al (2023) | Xanthohumol improves cognitive impairment by regulating miRNA-532-3p/Mpped1 in ovariectomized mice | Executive functions not an outcome |
| Maldonado-Lasuncion et al. (2019) | Aging-Related Changes in Cognition and Cortical Integrity are Associated With Serum Expression of Candidate MicroRNAs for Alzheimer Disease | Executive functions not an outcome |
| Mei et al (2023) | Lowering hippocampal miR-29a expression slows cognitive decline and reduces beta-amyloid deposition in 5xFAD mice | Pre-print |
| Mengel-From et al. (2018) | Circulating microRNAs disclose biology of normal cognitive function in healthy elderly people - a discovery twin study | Executive functions not an outcome |
| Nguyen and Kim (2022) | The role of mixed B vitamin intakes on cognitive performance: Modeling, genes and miRNAs involved | No primary microRNA data |
| Piergiorge (2023) | Multi-layered transcriptomic analysis reveals a pivotal role of FMR1 and other developmental genes in Alzheimer's disease-associated brain ceRNA network | No primary microRNA data |
| Pons-Espinal et al (2019) | MiR-135a-5p Is Critical for Exercise-Induced Adult Neurogenesis | Executive functions not an outcome |
| Puigoriol-Illamola et al (2020) | Chronic Mild Stress Modified Epigenetic Mechanisms Leading to Accelerated Senescence and Impaired Cognitive Performance in Mice | Executive functions not an outcome |
| Ragusa et al (2020) | Potential Associations Among Alteration of Salivary miRNAs, Saliva Microbiome Structure, and Cognitive Impairments in Autistic Children | Executive functions not an outcome |
| Rovcanin et al (2022) | Inflammation-Related microRNAs-146a and -155 Are Upregulated in Mild Cognitive Impairment Subjects Among Older Age Population in Montenegro | Executive functions not an outcome |
| Sadlon et al (2023) | Association of Blood MicroRNA Expression and Polymorphisms with Cognitive and Biomarker Changes in Older Adults | Executive functions not an outcome |
| Salama et al (2019) | Risk Factors Associated with Mild Cognitive Impairment among Apparently Healthy People and the Role of MicroRNAs | Executive functions not an outcome |
| Sampath et al (2023) | Sex differences in cognitive impairment after focal ischemia in middle-aged rats and the effect of iv miR-20a-3p treatment | Executive functions not an outcome |
| Shi et al (2021) | Phosphodiesterase-4D Knockdown in the Prefrontal Cortex Alleviates Memory Deficits and Synaptic Failure in Mouse Model of Alzheimer's Disease | No microRNA analysis |
| Shima et al (2023) | Light-intensity exercise improves memory dysfunction with the restoration of hippocampal MCT2 and miRNAs in type 2 diabetic mice | Executive functions not an outcome |
| Tsamou et al (2023) | A Candidate microRNA Profile for Early Diagnosis of Sporadic Alzheimer's Disease | Executive functions not an outcome |
| Valiulyte et al (2022) | Associations of miR-181a with Health-Related Quality of Life, Cognitive Functioning, and Clinical Data of Patients with Different Grade Glioma Tumors | Cancer patients’ population |
| Wei C et al. (2017) | MiR-7684-5p leads to surgery-induced cognitive decline in mice probably through the downregulation of SorLA | Executive functions not an outcome |
| Wei Z. et al (2020) | Environmental enrichment prevents Aβ oligomer-induced synaptic dysfunction through mirna-132 and hdac3 signaling pathways | Executive functions not an outcome |
| Xiao G. et al. (2021) | MicroRNA-455-5p/CPEB1 pathway mediates Aβ-related learning and memory deficits in a mouse model of Alzheimer's disease | Executive functions not an outcome |
| Xie G. et al (2022) | EVAtool: an optimized reads assignment tool for small ncRNA quantification and its application in extracellular vesicle datasets | Executive functions not an outcome |
| Yuan et al (2022) | Diagnostic performance of miR-21, miR-124, miR-132, and miR-200b serums in post-stroke cognitive impairment patients | Executive functions not an outcome |
| Zhang R. et al (2022) | Advanced Glycosylation End Products Induced Synaptic Deficits and Cognitive Decline Through ROS-JNK-p53/miR-34c/SYT1 Axis in Diabetic Encephalopathy | Executive functions not explicitly evaluated |
| Zhang Y. et al (2023) | Microglial exosomes alleviate intermittent hypoxia-induced cognitive deficits by suppressing NLRP3 inflammasome | Executive functions not an outcome |
| Zheng C. et al (2016) | Electroacupuncture Ameliorates Learning and Memory and Improves Synaptic Plasticity via Activation of the PKA/CREB Signaling Pathway in Cerebral Hypoperfusion | Executive functions not an outcome |
| Zheng D. et al (2022) | Screening of Human Circular RNAs as Biomarkers for Early Onset Detection of Alzheimer's Disease | Executive functions not an outcome |
| Zhou C et al (2022) | Genetic Deficiency of MicroRNA-15a/16-1 Confers Resistance to Neuropathological Damage and Cognitive Dysfunction in Experimental Vascular Cognitive Impairment and Dementia | Executive functions not an outcome |

eTable9. **Risk of bias assessment – human cohorts**

| **Study ID** | **SELECTION** | | | **COMPARABILITY** | **OUTCOME** | | |
| --- | --- | --- | --- | --- | --- | --- | --- |
|  | **ITEM 1** | **ITEM 2** | **ITEM 3** | **ITEM 5** | **ITEM 6** | **ITEM 7** | **ITEM 8** |
| Andrews 2017 |  |  |  |  |  |  |  |
| Aparicio 2021 |  |  |  |  |  | NA |  |
| Bijkerk 2022 |  |  |  |  |  | NA |  |
| Dypas 2023 |  |  |  |  |  |  |  |
| Gonzalez 2016 |  |  |  |  |  | NA |  |
| Islam 2014 |  |  |  |  |  | NA |  |
| Liu 2014 |  |  |  |  |  | NA |  |
| Park 2022 |  |  |  |  |  | NA |  |
| Potkin 2010 |  |  |  |  |  | NA |  |
| Schroeder 2014 |  |  |  |  |  | NA |  |

**Legend.** Point not achieved. Point achieved. NA: not applicable.

eTable10. **Risk of bias assessment – human case-control studies**

| **Study ID** | **SELECTION** | | | | **COMPARABILITY** | **OUTCOME** | |
| --- | --- | --- | --- | --- | --- | --- | --- |
|  | **ITEM 1** | **ITEM 2** | **ITEM 3** | **ITEM 4** | **ITEM 5** | **ITEM 6** | **ITEM 7** |
| Cosgrove 2017 |  |  |  |  |  |  |  |
| Gou 2020 |  |  |  |  |  |  |  |
| Han 2020 |  |  |  |  |  |  |  |
| Ignacio 2015 |  |  |  |  |  |  |  |
| Ma 2014 |  |  |  |  |  |  |  |
| O'Meara 2019 |  |  |  |  |  |  |  |
| Pan 2021 |  |  |  |  |  |  |  |
| Qi 2018 |  |  |  |  |  |  |  |
| Sun 2020 |  |  |  |  |  |  |  |
| Toyama 2023 |  |  |  |  |  |  |  |
| van Erp 2014 |  |  |  |  |  |  |  |
| Wang 2023 |  |  |  |  |  |  |  |
| Zhang 2023 |  |  |  |  |  |  |  |

**Legend.** Point not achieved. Point achieved. NA: not applicable.

eTable11. **Summary of the Network Analysis of the microRNAs’ gene targets**

| **Network ID** | **Nodes** | **Edges** | **Avg number of neighbors** | **Network Density** | **Top 10 gene Hubs (By highest degree)** |
| --- | --- | --- | --- | --- | --- |
| HSA - Group 1 | 943 | 1391 | 2.906 | 0.003 | MYC, HIF1A, SP1, TP53, CREB1, GABPA, MAX, MED1, NOTCH1, RUNX1T1 |
| HSA - Group 2 | 610 | 735 | 2.390 | 0.004 | NOTCH1, BCL2, CREB1, EP300, GATA6, IGF1R, MYC, PBX3, STAT3,TGFBR1 |
| HSA - Group 3 | 326 | 525 | 3.209 | 0.010 | AR, BRD4, CTCF, EGR1, EP300, FOXA1, KDM5B, MAX, MYC, RELA |
| MMU - Group 1 | 126 | 125 | - | 0.016 | All have a degree of 1 |
| MMU - Group 2 | 453 | 583 | 2.552 | 0.006 | MYC, JUN, BCL2, EP300, PPARG, STAT1, ZEB2, ZEB1, CTCF, EGR1 |
| MMU - Group 3 | 189 | 200 | 2.032 | 0.011 | CEBPB, SPI1, ETS1, FOS, IRF8, JUN, MAF, HDAC4, RBPJ, MYOD1 |
| MMU - Group 4 | 120 | 145 | 2.383 | 0.020 | CEBPB, NR3C1, EP300, RUNX2, RXRA, CREB1, CTCF, HNF1A, HNF4A, NOTCH1 |

**Legend.** HSA: Human; MMU: Mouse. Commonly found hubs are in bold.

eFigure2. **Risk of bias traffic light plot – Animal studies “SYRCLE tool”**

Legend. The Risk of Bias figures were created using the *robvis* online tool. ^5^

eFigure3. **Risk of bias summary plot – Animal studies “SYRCLE tool”**

eFigure4. **Risk of bias traffic light plot – Human Studies “RoB2 tool”**

**eTable11. Biological processes found after gene ontology enrichment analysis**

| **Groups** | **N° micro-RNAs entered** | **Enrich GO of brain enriched targets** |
| --- | --- | --- |
| Humans: Group 1 | 11  HSA-MIR-16-5P  HSA-MIR-200C-3P  HSA-MIR-29B  HSA-MIR-31-5P  HSA-MIR-3138  HSA-MIR-320E  HSA-MIR-374B-5P  HSA-MIR-4279  HSA-MIR-652-3P  HSA-MIR-9  HSA-LET-7I |  |
| Humans: Group 2 | 10  HSA-MIR-132  HSA-MIR-146A-5P  HSA-MIR-148A-3P  HSA-MIR-181A-5P  HSA-MIR-181B-5P  HSA-MIR-216B-3P  HSA-MIR-34C-5P  HSA-MIR-501-3P  HSA-MIR-504-5P  HAS-LET-7C-5P |  |
| Humans:  Group 3 | 7  HSA-MIR-1207-5P  HSA-MIR-1282  HSA-MIR-3201  HSA-MIR-3917  HSA-MIR-4734  HSA-MIR-520A-5P  HSA-MIR-663A |  |
| Rodents: Group 1 | 1  MMU-MIR-9-5P |  |
| Rodents: Group 2 | 20  MMU-MIR-132  MMU-MIR-133A-5P  MMU-MIR-146A-5P  MMU-MIR-148A-3P  MMU-MIR-152-3P  MMU-MIR-16-5P  MMU-MIR-181A-5P  MMU-MIR-183-5P  MMU-MIR-199A-5P  MMU-MIR-199B-3P  MMU-MIR-200B-3P  MMU-MIR-200C-3P  MMU-MIR-30E  MMU-MIR-34A  MMU-MIR-429-3P  MMU-MIR-708-5P  MMU-MIR-98-5P  MMU-LET-7I-5P  MMU-LET-7F-5P  MMU-LET-7G-5P |  |
| Rodents: Group 3 | 2 miR (3 entries)  MMU-MIR-155  MMU-MIR-155HG  MMU-MIR-206 |  |
| Rodents: Group 4 | 6  MMU-MIR-139-5P  MMU-MIR-190A  MMU-MIR-190B  MMU-MIR-222-3P  MMU-MIR-30A  MMU-MIR-31 |  |

# **eReferences**

1.	Hooijmans CR, Rovers MM, de Vries RB, Leenaars M, Ritskes-Hoitinga M, Langendam MW. SYRCLE's risk of bias tool for animal studies. *BMC medical research methodology* 2014; **14:** 43.

2.	Fabian-Jessing BK, Vallentin MF, Secher N, Hansen FB, Dezfulian C, Granfeldt A *et al.* Animal models of cardiac arrest: A systematic review of bias and reporting. *Resuscitation* 2018; **125:** 16-21.

3.	Eldridge S, Campbell M, Campbell M, Drahota DA, Giraudeau B, Higgins J *et al.* Revised Cochrane risk of bias tool for randomized trials (RoB 2.0). 2016.

4.	Deeks JJ HJ, Altman DG, Green S. Cochrane handbook for systematic reviews of interventions version 5.1. 0 (updated March 2011). Chapter 9.5.2.4. . *The Cochrane Collaboration* 2011.

5.	McGuinness LA, Higgins JPT. Risk-of-bias VISualization (robvis): An R package and Shiny web app for visualizing risk-of-bias assessments. *Research Synthesis Methods* 2020; **n/a**(n/a).
